# Zinc-dependent substrate-level phosphorylation powers *Salmonella* growth under nitrosative stress of the innate host response

**DOI:** 10.1371/journal.ppat.1007388

**Published:** 2018-10-26

**Authors:** Liam Fitzsimmons, Lin Liu, Steffen Porwollik, Sangeeta Chakraborty, Prerak Desai, Timothy Tapscott, Calvin Henard, Michael McClelland, Andres Vazquez-Torres

**Affiliations:** 1 Department of Immunology and Microbiology, University of Colorado School of Medicine, Aurora, CO, United States of America; 2 Department of Microbiology and Molecular Genetics, University of California Irvine School of Medicine, Irvine, CA, United States of America; 3 Research Service, Veterans Affairs Eastern Colorado Health Care System, Denver, CO, United States of America; Vanderbilt University, UNITED STATES

## Abstract

The metabolic processes that enable the replication of intracellular *Salmonella* under nitrosative stress conditions engendered in the innate response of macrophages are poorly understood. A screen of *Salmonella* transposon mutants identified the ABC-type high-affinity zinc uptake system ZnuABC as a critical determinant of the adaptation of *Salmonella* to the nitrosative stress generated by the enzymatic activity of inducible nitric oxide (NO) synthase of mononuclear phagocytic cells. NO limits the virulence of a *znuB* mutant in an acute murine model of salmonellosis. The ZnuABC transporter is crucial for the glycolytic function of fructose bisphosphate aldolase, thereby fueling growth of *Salmonella* during nitrosative stress produced in the innate response of macrophages. Our investigations demonstrate that glycolysis mediates resistance of *Salmonella* to the antimicrobial activity of NO produced in an acute model of infection. The ATP synthesized by substrate-level phosphorylation at the payoff phase of glycolysis and acetate fermentation powers the replication of *Salmonella* experiencing high levels of nitrosative stress. In contrast, despite its high potential for ATP synthesis, oxidative phosphorylation is a major target of inhibition by NO and contributes little to the antinitrosative defenses of intracellular *Salmonella*. Our investigations have uncovered a previously unsuspected conjunction between zinc homeostasis, glucose metabolism and cellular energetics in the adaptation of intracellular *Salmonella* to the reactive nitrogen species synthesized in the innate host response.

## Author summary

Microbial pathogens are exposed to multiple antimicrobial defenses during their associations with host cells. Nitric oxide generated in the innate response exerts widespread antimicrobial activity against a variety of pathogenic microorganisms. Nitric oxide has high affinity for metal groups of terminal cytochromes of the respiratory chain, and thus nitrosative stress exerts extreme deleterious actions against the cellular energetics that rely on oxidative phosphorylation. Intracellular *Salmonella* have resolved this dilemma by satisfying a significant portion of their energetic demands via substrate level phosphorylation in the payoff phase of glycolysis and acetate fermentation. A high affinity zinc uptake system promotes antinitrosative defense of intracellular *Salmonella* by in great part supporting the enzymatic activity of an essential enzyme in the preparatory phase of glycolysis. Our research provides novel insights into the metabolic and energetic adaptations that allow a bacterial pathogen to thrive in the midst of the innate host response of vertebrate cells.

## Introduction

Many of the more than 2,500 serovars of *Salmonella enterica* cause gastrointestinal or disseminated infections in millions of people and livestock every year [1, 2]. Reactive nitrogen species synthesized abiotically in the gastric lumen and the extreme acidity of the stomach constitute a formidable barrier to most microorganisms. However, *Salmonella* and other enteropathogens can endure these innate host defenses [3]. In the gastrointestinal tract, *Salmonella* competes for nutrients and space with members of the resident microbiota and, aided by the cytoskeletal remodeling induced by effectors of the *Salmonella* pathogenicity island-1 (SPI-1) type-III secretion system, forces its way into enterocytes and M cells of Peyer’s patches. The *Salmonella* SPI-1 effector SopB activates the transcription of *Nos2*-encoded inducible nitric oxide synthase (iNOS) long after invasion [4, 5]. Transcription of *Nos2* is independently activated in mononuclear phagocytic cells in response to lipopolysaccharide, fimbriae or porins imbedded in *Salmonella*’s cell envelope [6]. The iNOS flavohemoprotein synthesizes nitric oxide (NO) from the guanidino group of L-arginine and molecular oxygen (O_2_) [6–8]. The diatomic gas NO combines with O_2_, superoxide anion, iron and low-molecular weight thiols, generating a plethora of reactive nitrogen species that are endowed with vigorous antimicrobial activity [6].

By modifying thiol groups in redox active cysteines, the Feα of iron-sulfur clusters, and ferrous or ferric ions in heme cofactors, NO and its oxidative and nitrosative congeners exert cytostasis against *Salmonella* and various other microbial pathogens [6]. Reactive nitrogen species inhibit quinol oxidases, aconitase, pyruvate dehydrogenase, α-ketoglutarate dehydrogenase and dihydroxy acid dehydratase, thereby restricting oxidative phosphorylation, the tricarboxylic acid (TCA) cycle, and the biosynthesis of methionine, lysine, leucine, isoleucine and valine [9–11]. Nitrosative stress is often accompanied by demetallation and loss of protein function [6, 12, 13]. The released iron, manganese or zinc ions can further disrupt cellular functions by mismetallating metabolic enzymes [14]. The liberation of ferrous iron can also cause genotoxicity via the Fenton-catalyzed synthesis of hydroxyl and ferryl radicals [15]. Despite the many molecular targets poisoned by NO and its congeners, the antimicrobial activity of reactive nitrogen species varies widely against diverse intracellular and extracellular bacterial pathogens. Whereas *Pseudomonas aeruginosa*, *Mycobacterium tuberculosis*, and *Burkholderia spp*. are readily killed by NO [16–20], *Staphylococcus aereus* continuously replicates in the presence of nitrosative stress levels that are inhibitory to other microorganisms [21].

*S*. *enterica* shows intermediate phenotypes. Chemically-generated NO is bacteriostatic against this facultative intracellular enteropathogen [22], but intracellular *Salmonella* grow remarkably well in the presence of high fluxes of NO synthesized enzymatically by iNOS in the innate response of macrophages [23]. The flavohemoglobin Hmp, cytochrome *bd*, and low-molecular weight thiols are the main effectors of the antinitrosative toolbox of *Salmonella* [6, 9, 21, 24, 25]. In addition to these detoxification systems, the *Salmonella* pathogenicity island-2 type III secretion system helps *Salmonella* evade contact with iNOS-containing vacuoles [26]. Little is known about the metabolic adaptations that protect intracellular *Salmonella* against nitrosative stress produced by the host. Herein, we have exploited an unbiased Tn-seq approach to identify hitherto unknown antinitrosative defenses of *Salmonella*. Our investigations indicate that the high-affinity zinc transporter ZnuABC enables ATP synthesis via substrate-level phosphorylation in glycolysis and acetate fermentation, thereby sustaining *Salmonella* growth during the nitrosative stress that is generated in the innate host response of macrophages.

## Results

### Diversification of carbon sources minimizes the anti-*Salmonella* activity of NO

**Fig 1:**
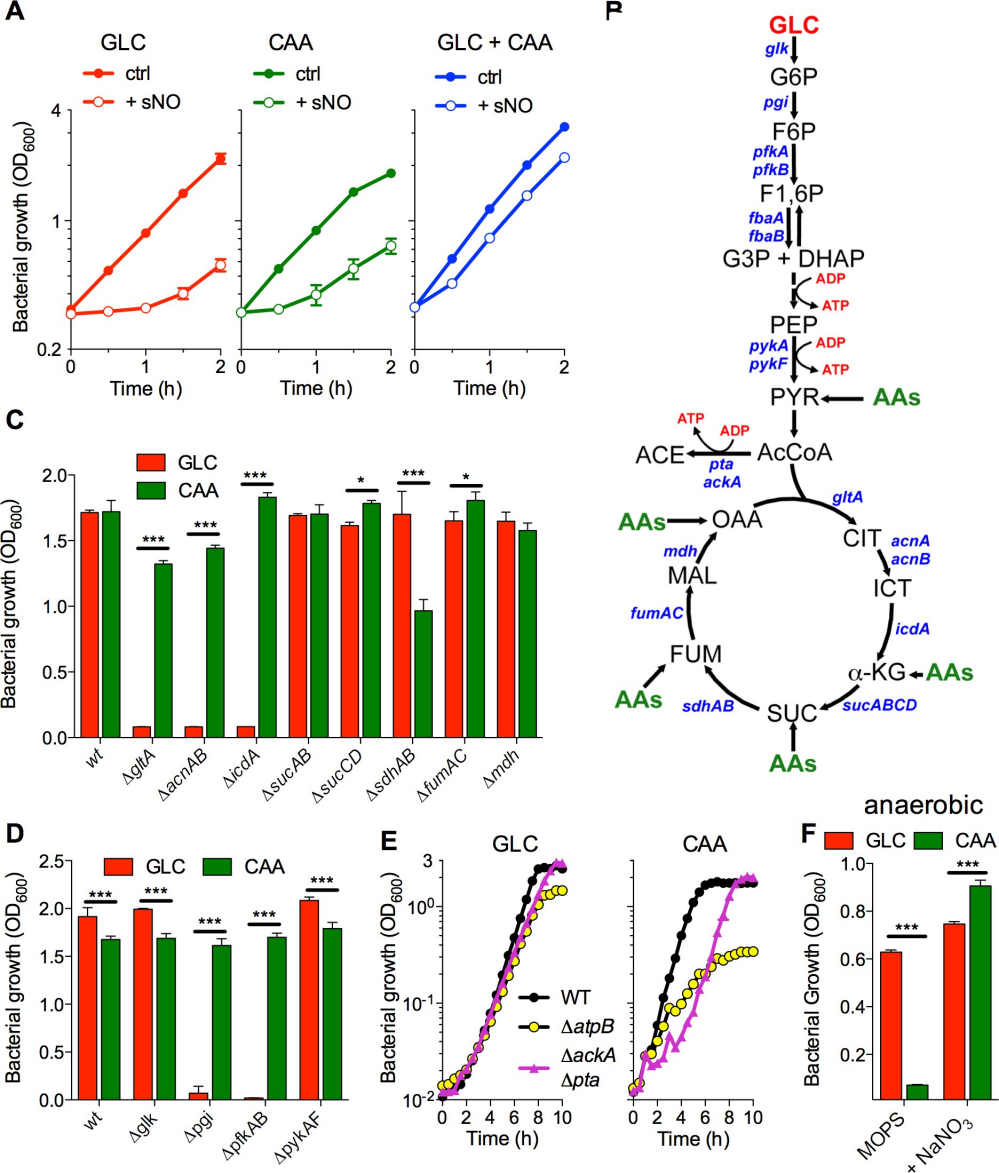
Effects of carbon sources on resistance of *Salmonella* to NO. (A) Growth of *Salmonella* treated (+ sNO) or untreated (ctrl) with 750 μM of spermine NONOate in MOPS minimal media supplemented with either glucose (GLC), casamino acids (CAA), or both carbon sources (GLC + CAA) (N = 4, mean ± S.E.M.). (B) Depiction of genes in glycolysis and tricarboxylic acid cycle (TCA) targeted for mutation. Sites of entry of GLC and CAA into glycolysis and TCA are shown. Steps of ATP synthesis by substrate-level phosphorylation and *fbaAB* genes encoding fructose bisphosphate aldolase are also indicated. Growth of *Salmonella* harboring mutations in TCA (C) or glycolysis (D) after 20 h of growth in MOPS minimal media supplemented with GLC or CAA (N = 3, mean ± S.E.M.). (E) Growth of wild-type (WT), Δ*atpB*, and Δ*ackA* Δ*pta Salmonella* in MOPS supplemented with GLC or CAA (N = 4, mean ± S.E.M.). (F) Anaerobic growth of *Salmonella* in MOPS media supplemented with 50 mM NaNO_3_ (N = 4, mean ± S.E.M.). *, *p* < 0.05; ***; *p* < 0.001.

NO exerts potent bacteriostasis against *Salmonella* in culture media, but this diatomic radical is well tolerated by intracellular *Salmonella*. In contrast to specialized bacterial pathogens that have undergone genomic and metabolic reduction [27], nontyphoidal *Salmonella* such as serovar Typhimurium can catabolize numerous carbon sources during their association with mammalian hosts [28]. Given the pressure NO exerts on central metabolism [9, 10, 12, 29], we investigated whether the availability of diverse carbon sources influences the tolerance of *Salmonella* to NO. The addition of the NO donor spermine NONOate induced a similarly prolonged lag phase in *Salmonella* grown in glucose or casamino acids (**Fig 1A**). The combination of casamino acids and glucose prevented most of the bacteriostasis associated with NO treatment (**Fig 1A**, **S1 Fig**). Similar trends were noted when *Salmonella* were challenged with the NO donors S-nitrosoglutathione (GSNO) or diethylenetriamine-NONOate (DETA NONOate) (**S1 Fig**). These findings indicate that *Salmonella* recovers rapidly from NO-induced cytotoxicity as long as it can gain access to diverse carbon sources. As suggested earlier [10, 11, 29], lysine, methionine and branch chain amino acids may allow *Salmonella* to circumvent NO-induced amino acid functional auxotrophies associated with the inactivation of lipoamide-dependent lipoamide dehydrogenase and dihydroxy acid dehydratase. However, the mechanisms by which glucose strengthens the antinitrosative defenses of *Salmonella* have not been determined yet.

To gain insights into the metabolic pathways by which glucose and casamino acids power the antinitrosative defenses of *Salmonella*, we examined the growth of mutants in glycolysis, acetate fermentation and TCA in glucose or casamino acids media (**Fig 1B**). Electron transport chain mutants were also tested. The inability of Δ*pgi*, Δ*pfkAB*, Δ*gltA*, Δ*acnAB and* Δ*icdA* mutants to replicate in glucose indicate that glycolysis and the oxidative branch of the TCA cycle support *Salmonella* growth in this hexose (**Fig 1C and 1D**). A Δ*pfkAB Salmonella* strain lacking both isoforms of the glycolytic enzyme phosphofructokinase grew in glycerol (**S1 Fig**), demonstrating that this mutant can grow in carbon entering glycolysis below fructose-1,6-bisphosphate. Δ*atpB* and Δ*ackA* Δ*pta* deletion mutants thrived in glucose, suggesting that ATP formed via oxidative phosphorylation or acetate fermentation is largely dispensable for growth of *S*. Typhimurium in glucose (**Fig 1E**). Aside from the minor growth defect of Δ*sdhAB Salmonella*, glycolytic and TCA cycle mutants grew robustly in casamino acids (**Fig 1C and 1D**). Casamino acids, a mixture of small peptides and amino acids, enter central metabolism at different steps in glycolysis and the TCA cycle (**Fig 1B**), likely explaining why single glycolytic or TCA cycle mutants flourished in this carbon source. *Salmonella*, however, required oxidative phosphorylation and acetate fermentation for optimal growth on casamino acids (**Fig 1E**). Moreover, casamino acids supported *Salmonella* growth as long as the terminal electron acceptors O_2_ or NO_3_^-^ were available. Conversely, glucose fueled *Salmonella* growth in the absence of O_2_ or NO_3_^-^ (**Fig 1F**). Cumulatively, this research indicates that glucose and casamino acids energize *Salmonella* growth by engaging substrate-level and oxidative phosphorylation, respectively. Our data also indicate that the metabolic diversification associated with glycolysis, acetate fermentation, TCA cycle and the electron transport chain yields a population of *Salmonella* that is highly immune to NO.

### Importance of metabolism in resistance of *Salmonella* to nitrosative stress

**Fig 2:**
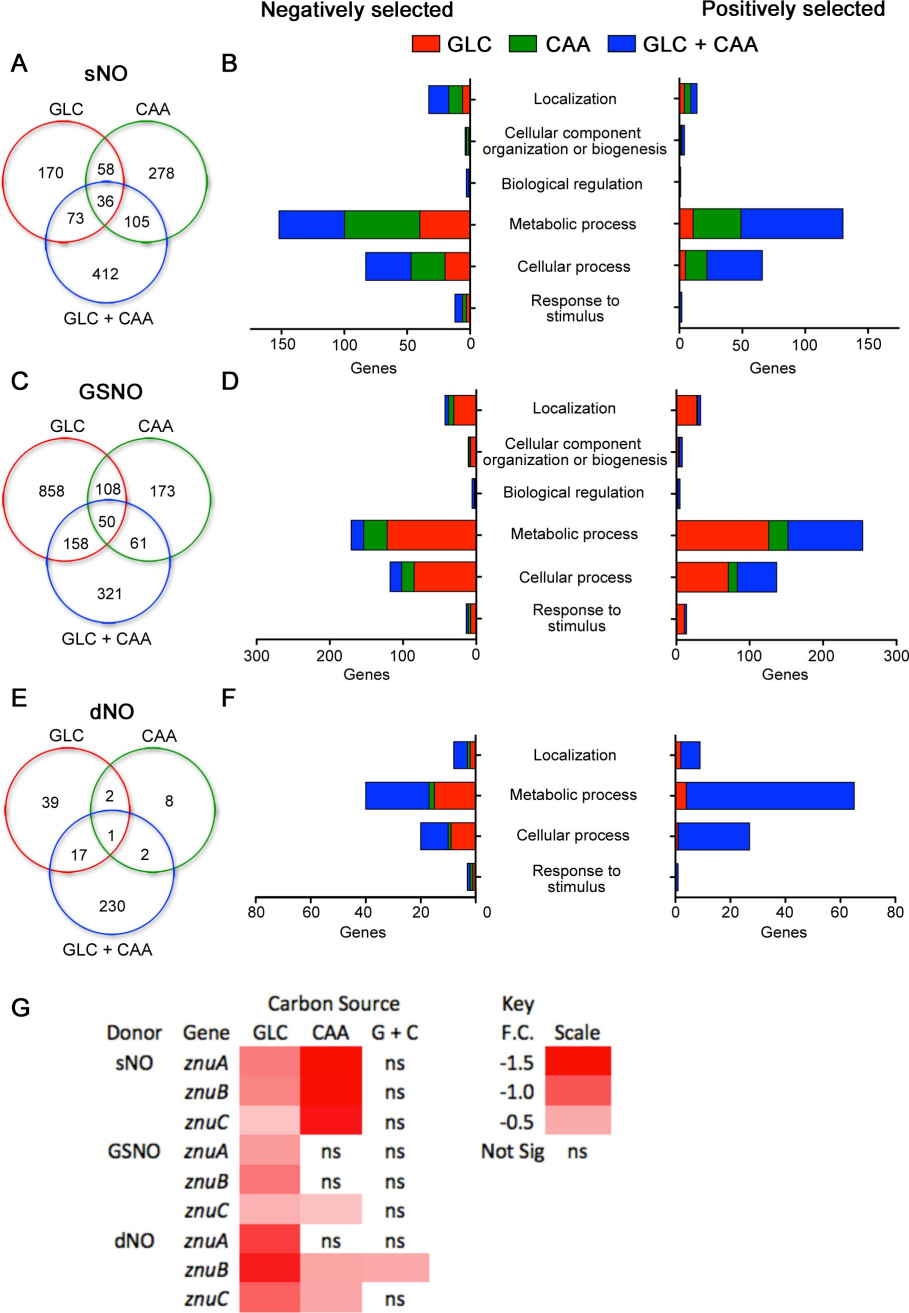
Selection of *Salmonella* transposon mutants by nitrosative stress. A library of 140,000 *Salmonella* transposon mutants grown in MOPS minimal media supplemented with either glucose (GLC), casamino acids (CAA), or glucose and casamino acids (GLC + CAA) were challenged with 750 μM of spermine NONOate (sNO), 5 mM S-nitrosoglutathione (GSNO) or 5 mM DETA NONOate (dNO) for 20 h. Genomic DNA was extracted, indexed by PCR, and analyzed by deep-sequencing. Gene mutations that showed differential survival with a false discovery rate < 10% were sorted into overlapping groups by media, and the number of common genes were plotted in Venn diagrams (A, C, and E). Positively or negatively selected genes for each NO donor and media were sorted by the Panther Gene Ontology program into biological process categories (B, D, and F). (G) Heat map of log_2_ fold changes (F.C.) of transposons in *znuABC* genes after NO treatment; ns: not statistically significant.

To gain further insights into the mechanisms by which nutritional diversity promotes antinitrosative defenses in *Salmonella*, we chose an unbiased transposon-based approach. A library of barcoded transposon mutants was separately challenged with the NO donors spermine NONOate, GSNO, or DETA NONOate in MOPS minimal media supplemented with glucose, casamino acids, or a combination of glucose and casamino acids. When the results were corrected to the media alone and the false discovery rate was set to < 10%, spermine NONOate, GSNO, and DETA NONOate exerted selectable advantages or disadvantages to 1132, 1729, and 299 genes, respectively. Differentially selected loci (**S1 Table**) were sorted into Venn diagrams (**Fig 2A, 2C and 2E**), and the annotated genes were converted into Go-terms. Panther Pathway analysis indicated that NO exerts the greatest negative and positive selection on loci related to metabolic processes (**Fig 2B, 2D and 2F**).

There were more common positively or negatively selected genes within each NO donor than within each medium (**S2 Table and S3 Table**). The fast releasing NO donor spermine NONOate selected against mutants in genes related to glycolysis (*pgk*, *pfkA*, *gpmA*), Tat-mediated secretion (*tatABC*), cell division (*zipA*, *ftsNL*) or proteostasis (*hflKCX*, *hslVU*, *clpA*), whereas mutations in SPI-1 (*invAFH*, *pipB2*, *sipA*) generated a growth advantage. As expected, mutations in the dipeptide uptake system (*dppABCDFG*), which transports S-nitrosoglutamyl cysteine across the cytoplasmic membrane [22], were positively selected in GSNO-treated *Salmonella*. The transnitrosating agent GSNO also enriched for mutations in purine (*purFH*) and cysteine (*cysCEFGQ*) biosynthesis, but selected against mutants in molybdopterin biosynthesis (*moaACDE*, *moeAB*). The slow NO donor DETA NONOate imposed a weaker selective pressure than spermine NONOate or GSNO. Mutations in the high-affinity, ABC-type, Zn^2+^ transporter (*znuABC*) and biotin biosynthesis (*bioABCDFH*) were negatively selected by DETA NONOate.

Genes were also grouped by medium (**S3 Table**). The NO donors tested selected for or against 11 (*e*.*g*., *znuABC*, *thiCF*, *nadC*) or 126 genes in *Salmonella* grown in glucose or casamino acids, respectively. Many of the positively selected transposon mutants in casamino acid-containing medium were deficient in glyoxylate shunt (*aceABK*), or biosynthesis of amino acids (*argABCEGI*, *thrBC*), thiamine (*thiCDFGH*), and lipopolysaccharide (*rfABGIJKLPZ*, *rfbABCDIKMNP*, *rfc*, *arnAT*, *basRS*, *pmrDFJLM*), suggesting that expression of these genes imposes a considerable metabolic burden when diverse nutrient sources are available to *Salmonella* undergoing nitrosative stress. As expected, NO negatively selected against mutations in *hmpA* and *gshAB* encoding the known antinitrosative defenses flavohemoglobin and glutathione synthase, respectively (**S4 Table**) [24, 25]. In addition, mutants in efflux and acquisition of Zn^2+^ (*zntA*, *znuC*, respectively), and modification of tRNAs (*cmoB*) were at a disadvantage when exposed to NO in casamino acids.

Overall, there were very few mutations that were consistently deleterious in most experimental conditions tested. Mutations in genes encoding the high-affinity, ABC-type, Zn^2+^ uptake system ZnuABC were the strongest exception. ZnuA is the zinc-binding, periplasmic cassette; ZnuB comprises the membrane-spanning permease; and ZnuC is the cytosolic ATPase that fuels uptake of this divalent cation [30]. Genetically, *znuA* is divergently transcribed from the *znuCB* operon [31]. Henceforth, this locus will be referred to as *znuABC*. Although transposon interruptions of either *znuA*, *znuB*, or *znuC* were under negative selection in most of the screen conditions tested, the disadvantage of the *znuA*, *znuB* and *znuC* mutants was most evident in glucose (**Fig 2G, S5 Table**), suggesting that Zn^2+^ uptake is particularly important in the antinitrosative defenses of glycolytic bacteria. However, not all components of zinc uptake contributed equally to the antinitrosative defenses of *Salmonella*, because NO did not exert significant pressures on *zntB*, *zitB*, or *zupT* genes encoding low-affinity Zn^2+^ transporters (**S6 Table**). NO did not seem to exert negative selection against *sodCI* or *sodCII* mutants deficient in copper-zinc superoxide dismutases (Cu-Zn SODs), which have been involved in resistance to phagocyte NADPH oxidase and iNOS [32].

### The *znuABC*-encoded Zn^2+^ uptake system protects *Salmonella* against the nitrosative stress engendered in the innate host response

**Fig 3:**
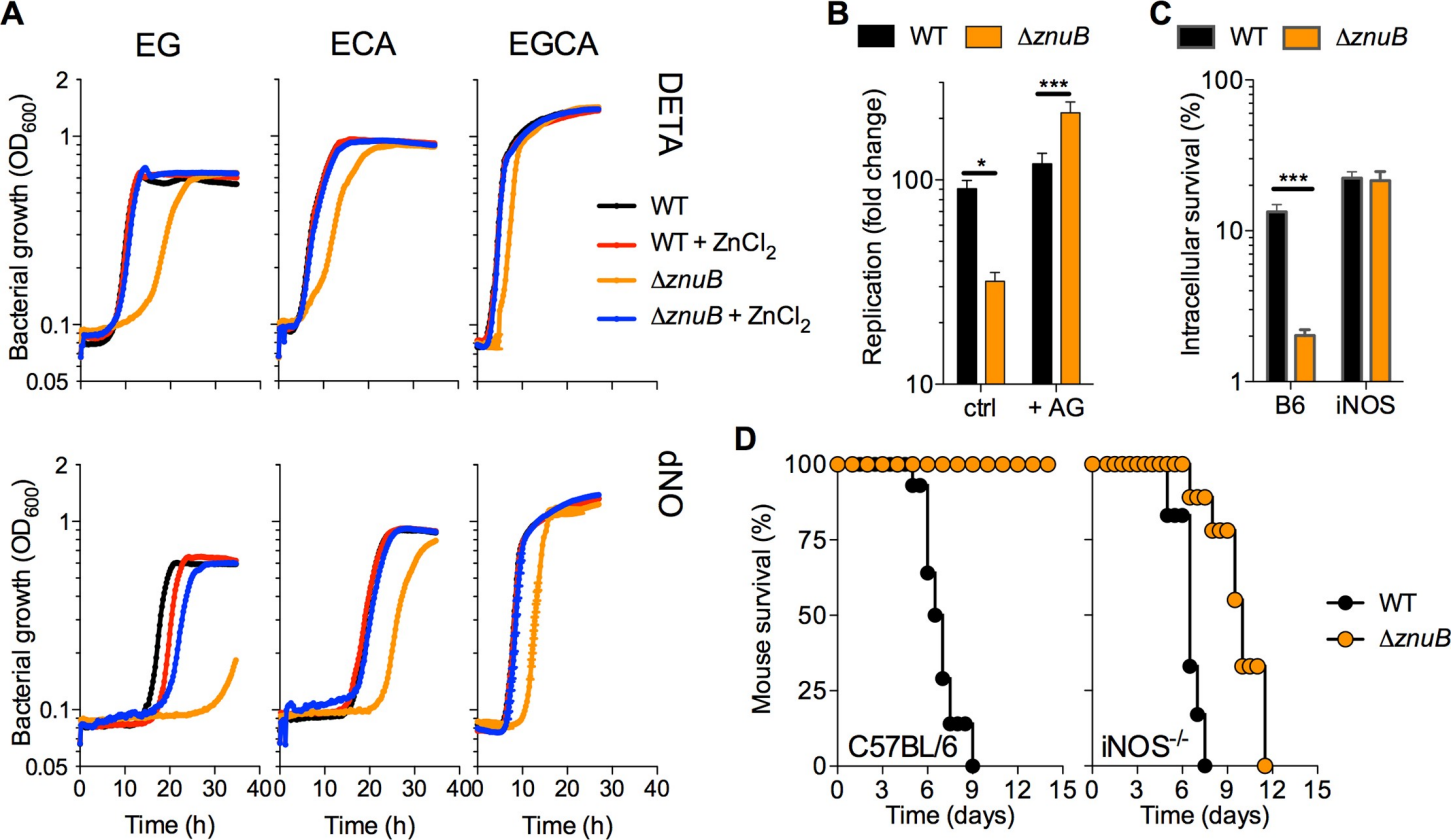
*Salmonella* require *znuB* to recover from nitrosative stress and cause disease. (A) Growth of wild-type (WT) and Δ*znuB Salmonella* after challenge with 5 mM diethylenetriamine (DETA) or 5 mM DETA NONOate (dNO) in E salts minimal media supplemented with glucose (EG), casamino acids (ECA), or glucose and casaminmo acids (EGCA). Where indicated, media were supplemented with 5 μM ZnCl_2_ (+ZnCl_2_) (N = 5 to 10, mean). (B) Intracellular growth of WT and Δ*znuB Salmonella* after 20 h of culture in J774 macrophage-like cells. Select samples were treated with 500 μM of the iNOS inhibitor aminoguanidine (AG) (N = 16, mean ± S.E.M.). (C) Intracellular survival of *Salmonella* in periodate-elicited macrophages from C57BL/6 or iNOS^-/-^ mice (N = 5, mean ± S.D.) * and ***, *p* < 0.05 and <0.001, respectively, as determined by two-way ANOVA. (D) Survival of C57BL/6 or iNOS deficient (iNOS^-/-^) mice infected *i*.*p*. with approximately 100 CFU of wild-type (WT) or Δ*znuB Salmonella* (N = 7–9 mice). *p* < 0.0001 and 0.001 for C57BL/6 and iNOS^-/-^, respectively, as determined by logrank analysis.

We evaluated in more detail the role that the high-affinity Zn^2+^ uptake system ZnuABC plays in resistance to NO generated chemically *in vitro* or enzymatically in the innate response of macrophages and mice. Compared to wild-type controls, Δ*znuB Salmonella* required longer times to enter exponential growth in all media examined (**Fig 3A, S2 Fig**). The Δ*znuB* mutant also had longer doubling times in glucose than wild-type controls (**S2 Fig**), further reinforcing the idea that zinc plays an essential role in glycolysis. The addition of 5 μM ZnCl_2_ restored normal growth to Δ*znuB Salmonella*, whereas the NO donor DETA NONOate exacerbated the growth defects of Δ*znuB Salmonella* in all carbon sources tested (**Fig 3A, S2 Fig**). Expression of a *znuB* allele in trans restored growth of Δ*znuB Salmonella* in EG minimal media to wild-type levels and it prevented the hypersusceptibility of this mutant to nitrosative stress (**S2 Fig**). Isogenic Δ*znuA* and Δ*znuC Salmonella* mutants also displayed hypersusceptibility to DETA NONOate (**S2 Fig**), indicating that mutations in any of the subunits that encode the high affinity ZnuABC zinc uptake system predispose *Salmonella* to nitrosative stress. In contrast, Δ*zur Salmonella* was as resistant to DETA NONOate as wild-type controls (**S2 Fig**), perhaps reflecting derepression of *znuABC* transcription [30, 31]. Collectively, these investigations suggest that glycolysis is particularly reliant on Zn^2+^ and that zinc-starved *Salmonella* are particularly sensitive to the bacteriostatic actions of NO.

We examined whether the antinitrosative defenses associated with the ZnuABC system protect *Salmonella* against NO engendered in the innate response of mononuclear phagocytic cells. Compared to wild-type controls, Δ*znuB Salmonella* replicated poorly in NO-producing J774 cells (**Fig 3B, S2 Fig)**. Ectopic expression of the *znuB* gene reversed the growth defect of Δ*znuB Salmonella* (**S2 Fig**). Moreover, the addition of the iNOS inhibitor aminoguanidine substantially reduced NO production (**S2 Fig**), and greatly promoted intracellular growth of Δ*znuB Salmonella* (**Fig 3B**). The iNOS inhibitor L-NIL also promoted intracellular growth of Δ*znuB Salmonella* in J774 cells (**S2 Fig**). Neither aminoguanidine nor L-NIL affected growth of *Salmonella in vitro* (**S2 Fig**), suggesting that the improved growth of Δ*znuB Salmonella* in J774 cells treated with aminoguanidine or L-NIL cannot be explained by direct effects of these drugs on the bacteria but rather on the inhibition of host iNOS enzymatic activity. Accordingly, Δ*znuB Salmonella* were also more susceptible than wild-type controls to the antimicrobial activity derived from NO congeners synthesized in the innate response of periodate-elicited macrophages (**Fig 3C, S2 Fig**). We also examined whether the ZnuABC system is a key component of the antinitrosative arsenal of *Salmonella* in a model of acute infection. Wild-type *Salmonella* killed C57BL/6 and *iNOS*^*-/-*^ mice 9 and 7.5 days post infection, respectively (**Fig 3D**). Consistent with previous investigations [33], Δ*znuB Salmonella* was attenuated in C57BL/6 mice (*p* < 0.0001). Interestingly, Δ*znuB Salmonella* became virulent in *iNOS*^*-/-*^ mice, indicating that this Zn^2+^ acquisition system is an integral aspect of the antinitrosative defenses of *Salmonella*. iNOS^-/-^ mice infected with Δ*znuB Salmonella* live on average 4 days longer (*p* < 0.001) than those infected with wild-type controls, suggesting that zinc acquisition also participates in *Salmonella* virulence in ways that are independent of antinitrosative defense.

### The Zn^2+^ uptake system ZnuABC is needed for maximal fructose bisphosphate aldolase activity

**Fig 4:**
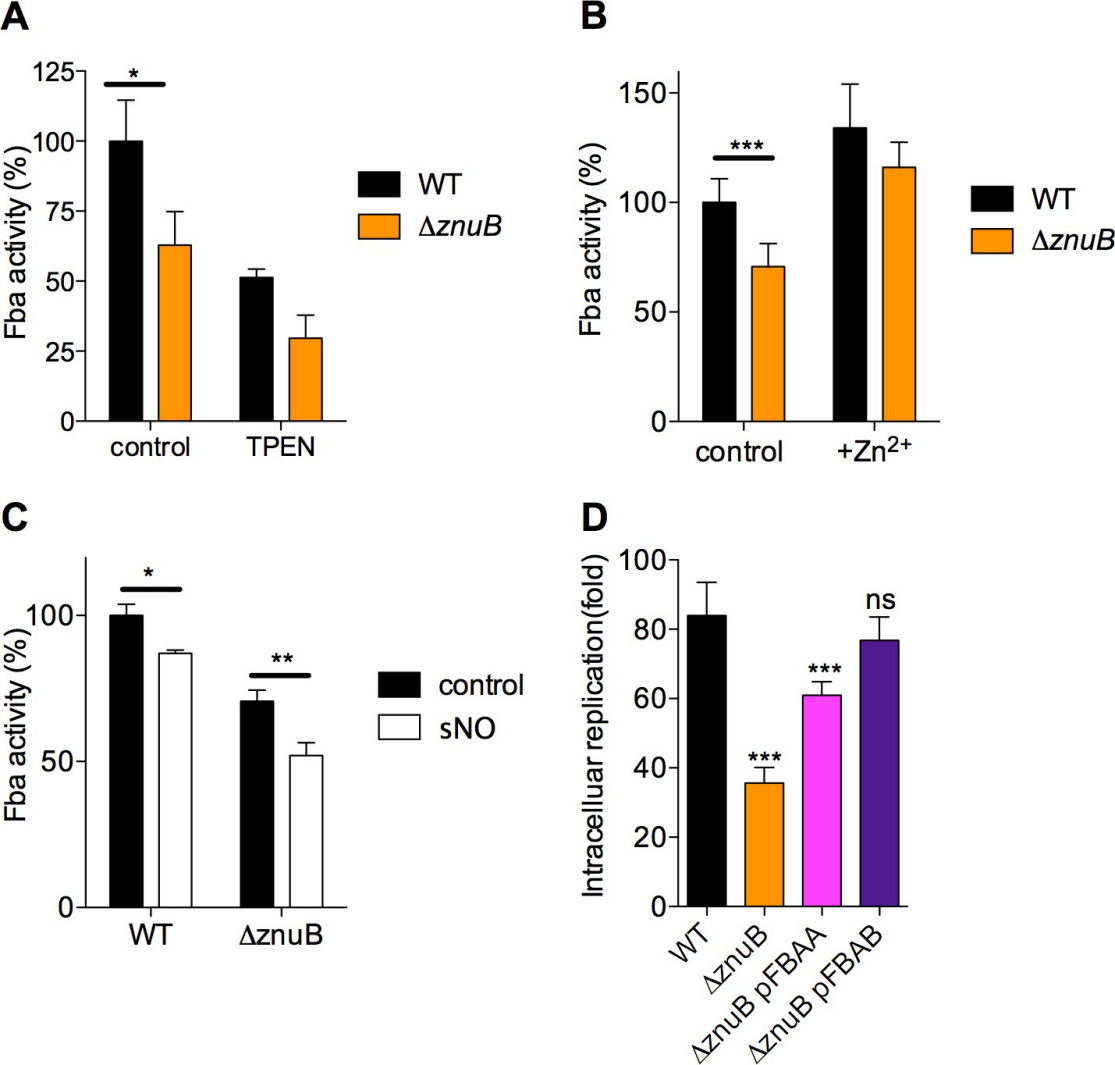
Fructose bisphosphatate aldolase content. (A) Fructose-1,6-bisphosphate aldolase (Fba) activity in *Salmonella* lysates. Where indicated, the lysates were treated with 100 μM of the zinc chelator TPEN. The Fba activity was normalized to WT samples. N = 12, mean ± S.E.M. (B) Effect of the supplementation of EG minimal media with 5 μM ZnCl_2_ on the Fba activity of log phase *Salmonella*. N = 8, mean ± S.D. (C) Fba activity in *Salmonella* treated for 5 min with 750 μM spermine NONOate. N = 8, mean ± S.D. *, **, *** *p* < 0.05, <0.01, <0.001, respectively, as determined by two-way ANOVA. (D) Growth of Δ*znuB Salmonella* after 16–20 h of culture in J774 cells. Where indicated, Δ*znuB Salmonella* were complemented with *fbaA* or *fbaB* genes heterologously expressed from the pBAD promoter. N = 8–16; mean ± S.D. ***, *p* < 0.001 as determined by one-way ANOVA.

In the preparative phase of glycolysis, fructose bisphosphate aldolase splits fructose-1,6-bisphosphate into dihydroxyacetone-phosphate and glyceraldehyde-3-phosphate. As is the case for *E*. *coli*, the *Salmonella* genome encodes for two isoforms of fructose-bisphosphate aldolase. The constitutively expressed class II fructose bisphosphate aldolase (encoded by *fbaA*) is zinc-dependent, whereas the expression of the zinc-independent class I isoform FbaB is induced in response to gluconeogenic substrates. Hence, in *E*. *coli* grown in glucose, 95–100% of the total fructose bisphosphate aldolase activity is contributed by the zinc-dependent class II fructose bisphosphate aldolase [34]. We noticed that Δ*znuB Salmonella* harbored about two thirds of the fructose bisphosphate aldolase enzymatic activity of wild-type controls (**Fig 4A**). Fructose bisphosphate activity was inhibited by the Zn^2+^-chelator N,N,N’,N’-tetrakis(2-pryidinylmethyl)-1,2-ethanediamine (**Fig 4A**). The addition of 5 μM ZnCl_2_ to the EG minimal media eliminated the differences in fructose bisphosphate aldolase activity between wild-type and Δ*znuB Salmonella* (**Fig 4B**). Together, these findings suggest that most differences in fructose bisphosphate aldolase content between wild-type and Δ*znuB Salmonella* are due to poor zinc uptake. NO inhibited (*p* < 0.05) by 10% the enzymatic activity of fructose bisphosphate aldolase in wild-type *Salmonella* (**Fig 4C**). NO appears to inhibit fructose bisphosphate aldolase to a greater extent in Δ*znuB Salmonella* (*p* < 0.01) than wild-type controls (*p* < 0.05).

We wondered if a 33% drop in fructose bisphosphate aldolase activity has a significant impact in the intracellular growth defect of Δ*znuB Salmonella*. To test this idea, Δ*znuB Salmonella* were complemented with the *fbaA* or *fbaB* genes, encoding zinc-dependent or–independent fructose bisphosphate aldolase isoforms, respectively (**Fig 4D**). Although intracellular growth was dramatically improved (*p* < 0.001), expression of *fbaA* gene did not fully restore growth of Δ*znuB Salmonella* to wild-type levels (*p* < 0.001 when Δ*znuB* pFBAA is compared to wild-type *Salmonella*). However, expression of the *fbaB* gene encoding the zinc-independent fructose bisphosphate aldolase isoform fully restored the intracellular growth of Δ*znuB Salmonella*. Together, these findings strongly indicate that the decreased enzymatic activity of fructose bisphosphate aldolase is a major contributor to the poor intracellular growth exhibited by Δ*znuB Salmonella*.

Zinc serves as cofactor for multiple proteins, including Cu-Zn SODs that detoxify superoxide anion in the periplasm. By limiting peroxynitrite formation from superoxide and NO, Cu-Zn SODs contribute to the antinitrosative defenses of *Salmonella* [32]. To assess if Δ*znuB Salmonella* has defects in detoxifying superoxide anion formed in the plasma membrane from the adventitious reduction of oxygen by NADH dehydrogenases [35], we monitored the formation of nitrotyrosine residues as a proxy of peroxynitrite. Wild-type and Δ*znuB Salmonella* harbored similar levels of nitrotyrosine after treatment with 500 μM spermine NONOate (**S3 Fig**). As predicted by the critical role NADH dehydrogenases play in the production of endogenous superoxide in *Salmonella*, a Δ*nuo* Δ*ndh* mutant showed a dramatic decrease in nitrotyrosine formation in response to spermine NONOate. Together, these findings suggest that Δ*znuB Salmonella* has normal SodC function, perhaps because, as it has been suggested earlier [36], metallation of apo-SodC is likely to occur in the periplasm.

### Glycolysis contributes to the antinitrosative defenses of *Salmonella*

**Fig 5:**
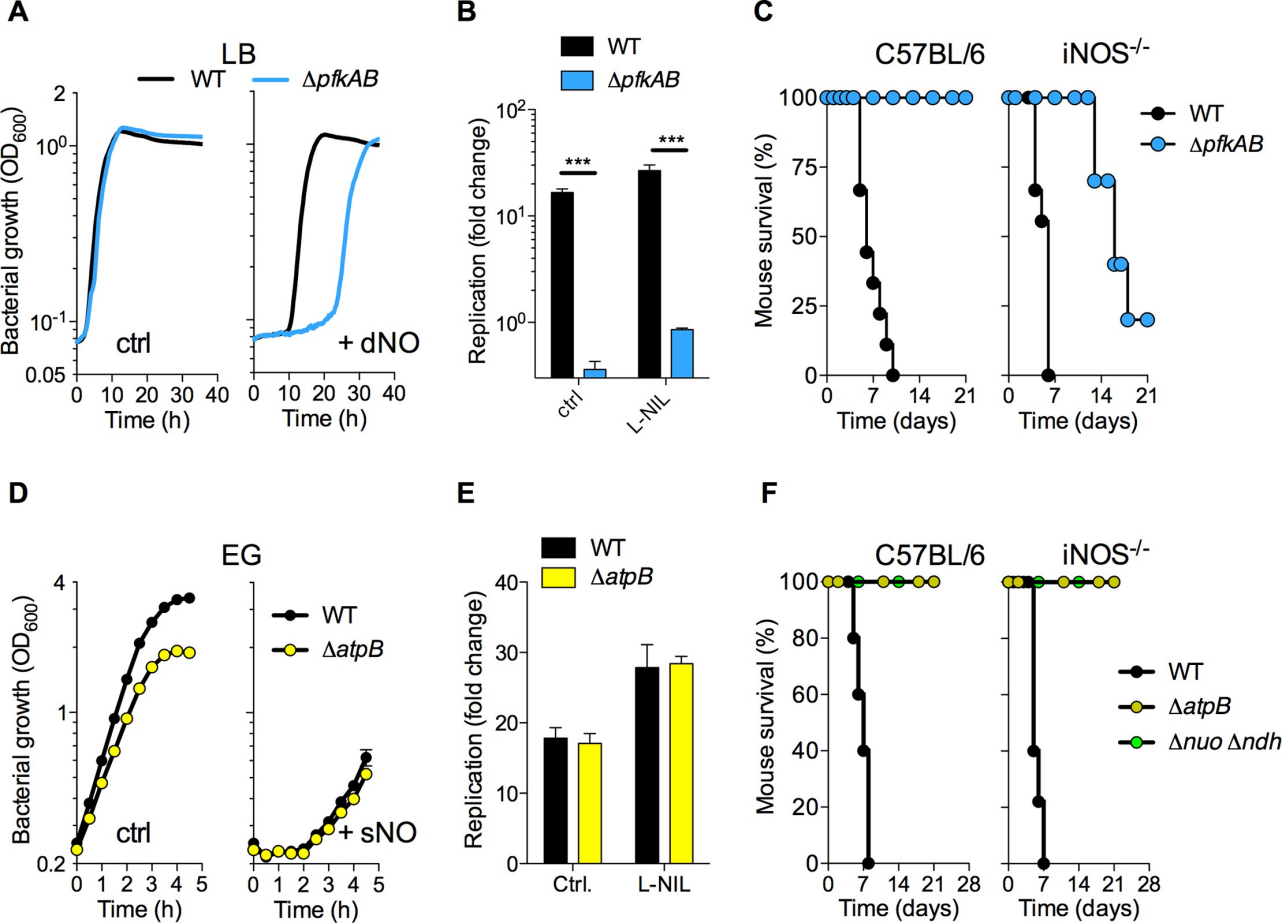
Glycolysis enhances the recovery of *Salmonella* from nitrosative stress. (A) Growth of wild-type (WT) and Δ*pfkAB Salmonella* in LB broth that was left untreated (ctrl) or treated with 500 μM DETA NONOate (dNO) (N = 5, mean). (B) Intracellular replication of WT and Δ*pfkAB Salmonella* 20 h after challenge of J774 macrophages. Select samples were treated with 500 μM of the iNOS inhibitor N6-(1-iminoethyl)-L-lysine (L-NIL) (N = 4–8, mean ± S.D.). ***, *p* < 0.001 as determined by two-way ANOVA. (C) Survival of C57BL/6 or iNOS deficient (iNOS^-/-^) mice after *i*.*p*. inoculation of 100 CFU of wild-type WT or Δ*pfkAB Salmonella*. (N = 9–10 mice). *p* < 0.01 for both C57BL/6 and iNOS^-/-^, as determined by logrank analysis. (D) Growth of the indicated *Salmonella* strains in EG minimal media. Where indicated, the specimens were treated with 750 μM spermine NONOate (+ sNO) (N = 4, mean ± S.E.M.). (E) Replication of *Salmonella* in J774 macrophages in the presence or absence of L-NIL (N = 4–8, mean ± S.D.). (F) Survival of C57BL/6 and iNOS^-/-^ mice after *i*.*p*. challenge with 100 CFU of WT, Δ*atpB*, or Δ*nuo* Δ*ndh Salmonella*. (N = 5 or 10 mice).

Given the role the ZnuABC uptake system plays in glycolysis and antinitrosative defense, we tested the contribution of glycolysis in the resistance of *Salmonella* to NO. The gene *fba*, encoding the zinc-dependent, fructose bisphosphate aldolase is an essential gene in *E*. *coli*. The lack of transposons in the gene indicate that it is also essential in *Salmonella*. Therefore, we tested the phenotypes of a glycolytic mutant deficient in the *pfkAB*-encoded phosphofructose kinase, and found that Δ*pfkAB Salmonella* suffered a prolonged lag phase when challenged with DETA NONOate (**Fig 5A, S4 Fig**). Consistent with previous reports [37, 38], Δ*pfkAB Salmonella* failed to replicate within macrophages (**Fig 5B**). Blockage of NO synthesis with the iNOS inhibitor N6-(1-iminoethyl)-L-lysine (L-NIL) partially rescued the intracellular growth defect of Δ*pfkAB Salmonella* in J774 macrophage cells (**Fig 5B, S4 Fig**). The intracellular replication defect and hypersuceptibility of Δ*pfkAB Salmonella* to DETA NONOate could be complemented by expression of *pfkA* or *pfkB* genes in trans (**S4 Fig**). The Δ*pfkAB Salmonella* strain was extremely attenuated in C57BL/6 mice but became virulent in iNOS^-/-^ mice (**Fig 5C**), strongly suggesting that glycolysis helps *Salmonella* to overcome the cytotoxicity of NO synthesized in the innate host response. As noted with Δ*znuB Salmonella*, iNOS^-/-^ mice succumbed more slowly to Δ*pfkAB Salmonella* infection than controls infected with wild-type *Salmonella* (*p* < 0.01), suggesting that glycolysis supports *Salmonella* virulence in NO-dependent and -independent ways.

In addition to glycolysis, oxidative phosphorylation can be a sizable source of ATP. Therefore, we also tested whether oxidative phosphorylation contributes to the antinitrosative defenses of *Salmonella*. We noted that a *Salmonella* strain deficient in the *atpB*-encoded subunit of ATP synthase recovered from nitrosative stress as efficiently as wild-type controls (**Fig 5D**). Furthermore, wild-type and Δ*atpB Salmonella* replicated to similar densities within NO-producing J774 macrophages (**Fig 5E, S4 Fig**). Given its excellent growth in J774 cells, we were surprised to find that Δ*atpB Salmonella* were not only severily attenuated in C57BL/6 mice but remained avirulent in iNOS^*-/-*^ mice. Similar results were obtained with a Δ*nuo* Δ*ndh* strain lacking both isoforms of the NADH dehydrogenase of the electron transport chain. Thus, the ATP synthase seems to be dispensable for antinitrosative defenses of intracellular *Salmonella*, but it is critical for *Salmonella* pathogenesis.

### Substrate-level phosphorylation sustains growth of intracellular *Salmonella* undergoing nitrosative stress

**Fig 6:**
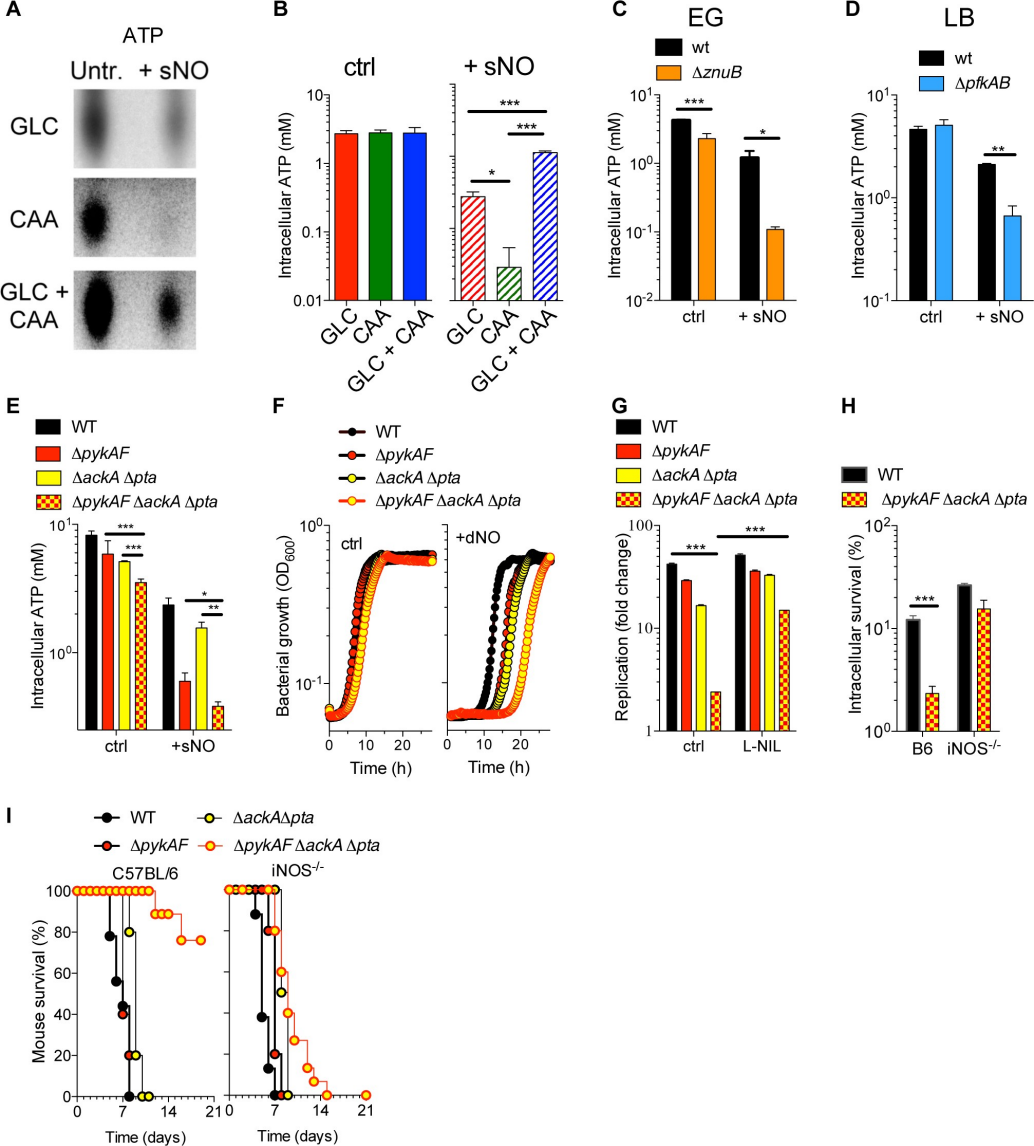
Effects of carbon source on the ATP pool of *Salmonella* undergoing nitrosative stress. (A) ATP was visualized after TLC analysis of ^32^P-labeled *Salmonella* grown in MOPS minimal media supplemented with either glucose (GLC) and/or casamino acids (CAA). When indicated, the cultures were treated with 750 μM spermine NONOate (sNO) for 5 min. Images are representative of experiments performed on at least two independent days. (B, C-E) The ATP pools were independently estimated with firefly luciferase. The bacteria were grown in EG (C and E) or LB broth (D). Select bacteria were treated with 750 μM sNO for 5 min (N = 6, mean ± S.E.M.). *, **, *** *p* < 0.05, 0.01, 0.001, respectively, as determined by one-way or two-way ANOVA. (F) Growth of the indicated *Salmonella* strains after treatment with 1 mM DETA NONOate (dNO) in EG media. (G) Intracellular growth of *Salmonella* in J774 cells 20 h after infection. Some of the specimens were treated with 500 μM L-NIL. (H) Intracellular survival of *Salmonella* in periodate-elicited macrophages from C57BL/6 or iNOS^-/-^ mice (N = 4–5, mean ± S.D.). ***, *p* < 0.001 as determined by two-way ANOVA. (I) Survival of C57BL/6 or iNOS^-/-^ mice after i.p. inoculation of 100 CFU of the indicated *Salmonella* strains. N = 9–15; * *p* < 0.05, WT vs. Δ*pykAF* Δ*ackA* Δ*pta* in C57BL/6 mice as determined by logrank analysis; all other comparisons to WT were not statistically significant.

Because substrate-level phosphorylation in glycolysis can be a sizable source of ATP, we examined the degree to which glucose utilization helps *Salmonella* maintain the ATP pool during nitrosative stress. Thin layer chromatography and independent firefly luciferase determinations indicated that spermine NONOate reduces the ATP pool in *Salmonella* growing in glucose or casamino acids (reductions of 90 vs. 99%, respectively) (**Fig 6A and 6B**). *Salmonella* growing in both glucose and casamino acids retained about 60% of the ATP pool after NO treatment (**Fig 6A and 6B**), indicating that access to diverse carbon sources that feed into glycolysis and the electron transport chain protects most of the ATP pool from the toxic actions of NO. The ATP pools were also measured in glycolytically-deficient Δ*znuB* and Δ*pfkAB Salmonella* and oxidative phosphorylation-deficient Δ*atpB Salmonella*. Because of its extreme growth defect in glucose, the Δ*pfkAB* mutant was tested in LB broth. Wild-type, Δ*atpB*, Δ*pfkAB* and Δ*znuB Salmonella* suffered about 3-, 5-, 8-, and 21-fold reductions, respectively, in the ATP pool upon NO challenge (**Fig 6C and 6D, S5 Fig**). Cumulatively, these findings suggest that zinc-dependent glycolysis is more effective than oxidative phosphorylation at preserving cellular energetics during periods of nitrosative stress.

The *pgk*-encoded phosphoglycerate kinase and *pykAF*-encoded pyruvate kinases generate ATP by substrate-level phosphorylation in the payoff phase of glycolysis. In addition, an ATP molecule is synthesized by the fermentation of acetyl-CoA to acetate by the *ackA*-encoded acetate kinase. Because *pgk* is an essential gene in *Salmonella*, we directed our attention to the *pykAF* and *pta*-*ackA* pathways. In increasing order, spermine NONOate depleted ATP from wild-type, Δ*ackA* Δ*pta*, Δ*pykAF* and Δ*pykAF* Δ*ackA* Δ*pta Salmonella* (**Fig 6E**), suggesting that glycolysis contributes most of the ATP synthesized by substrate-level phosphorylation in NO-treated *Salmonella* but that acetate fermentation is also an important source of ATP. Spermine NONOate inflicted more severe bacteriostasis to a Δ*pykAF* Δ*ackA* Δ*pta* mutant than Δ*pykAF* or Δ*ackA* Δ*pta* isogenic strains (**Fig 6F**), and NO produced in the innate response of J774 cells stunted the growth of Δ*pykAF* Δ*ackA* Δ*pta Salmonella* more severily than that of Δ*pykAF* or Δ*ackA* Δ*pta* controls (**Fig 6G**). The growth defects of Δ*pykAF* Δ*ackA* Δ*pta Salmonella* could be complemented by expression of the *ackA pta* operon in trans (**S5 Fig**). Δ*pykAF* Δ*ackA* Δ*pta Salmonella* were also more susceptible to the antimicrobial activity of iNOS expressed in the innate response of primary macrophages (**Fig 6H, S5 Fig**). Moreover, in contrast to the Δ*pykAF* or Δ*ackA* Δ*pta* parent strains, the Δ*pykAF* Δ*ackA* Δ*pta* mutant was attenuated in C57BL/6 mice (*p* < 0.05 as compared to wild-type *Salmonella*). Interestingly, Δ*pykAF* Δ*ackA* Δ*pta Salmonella* became as virulent as wild-type bacteria in iNOS^-/-^ mice (*p* = 0.5) (**Fig 6I**). Collectively, these investigations demonstrate that ATP synthesized by substrate-level phosphorylation in both glycolysis and acetate fermentation protects *Salmonella* against the cytotoxicity of NO produced in the innate response.

## Discussion

Zn^2+^, the second most abundant metal cofactor, provides structural, regulatory, antioxidant, and catalytic properties to diverse metalloproteins [39, 40]. Given its critical importance in bacterial cell physiology, mammalian hosts actively limit the bioavailability of Zn^2+^ to bacterial pathogens, thereby contributing to what is now known as nutritional immunity [41]. As a countermeasure, bacterial pathogens use high affinity Zn^2+^ transporters, such as ZnuABC or the Gram-positive AdcABC orthologue, to compete with the host for zinc [41]. Interestingly, *Salmonella* utilizes ZnuABC to compete for zinc with indigenous microbiota of the gut, and exploits this high-affinity uptake system to gain advantages in systemic sites and macrophages [33, 42]. The mechanisms underlying poor growth of Δ*znuB Salmonella* within macrophages have not been identified yet. Herein, we show that ZnuABC potentiates *Salmonella* pathogenesis by in part antagonizing the nitrosative stress generated in the innate response of macrophages and mice. Despite its widespread utilization in metabolism and multiple regulatory processes, the scarcity of zinc in Δ*znuB Salmonella* is particularly detrimental to zinc-dependent fructose bisphosphate aldolase in glycolysis as suggested by the complementation of the intracellular growth defects of Δ*znuB Salmonella* with the *fbaB* gene encoding zinc-independent fructose bisphosphate aldolase. Thus, not only does high affinity zinc uptake defend microbes against the metabolic stress associated with either nutritional immunity or competition with microbiota, but it also arms bacteria with the glycolytic flexibility needed to overcome the deficiencies in energetics that are triggered by the inhibition of cytochromes by NO of the innate response of professional phagocytes.

**Fig 7:**
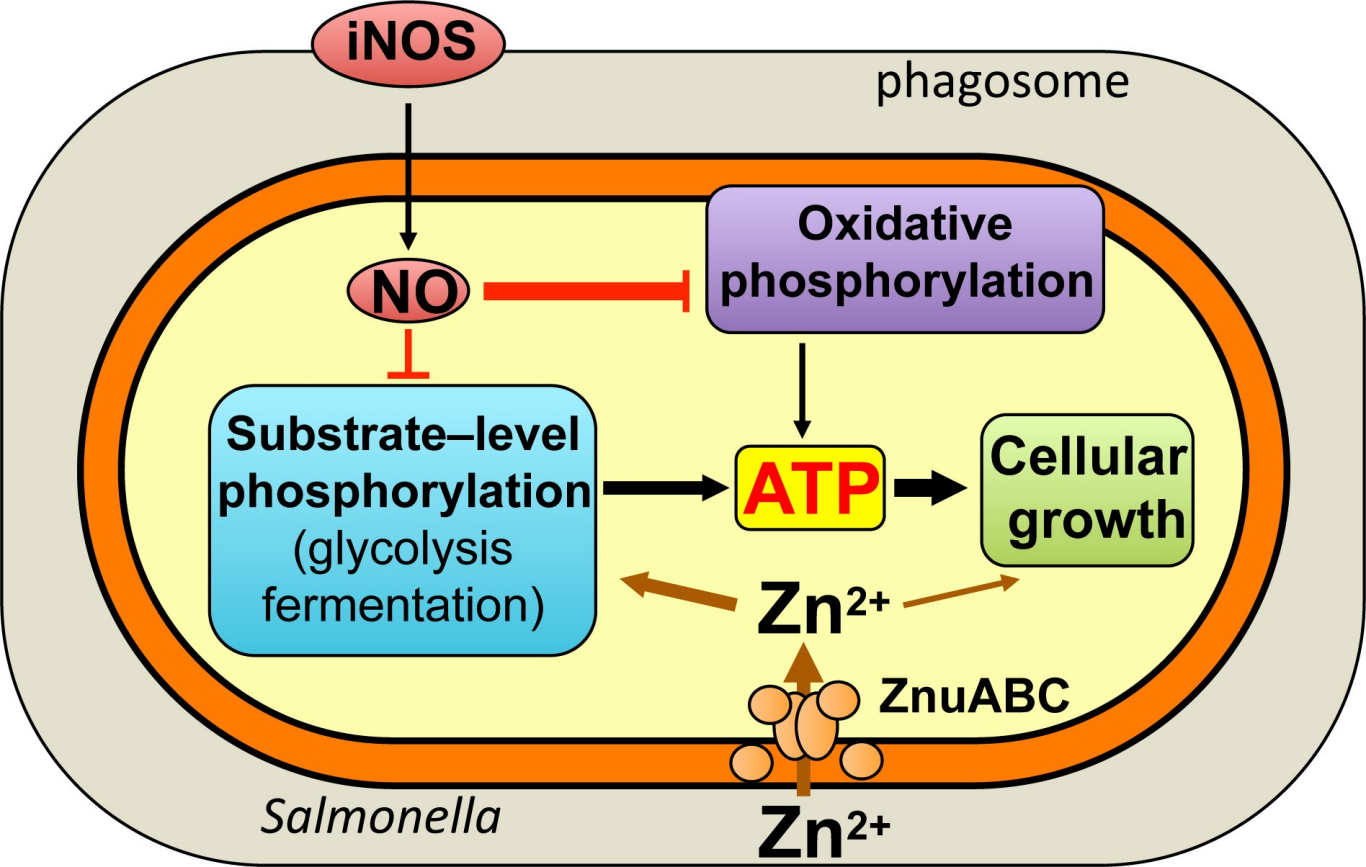
Model for zinc and energy metabolism in the antinitrosative defenses of *Salmonella*. ATP can be generated by substrate-level phosphorylation in glycolysis and acetate fermentation or oxidative phosphorylation in the electron transport chain. Nitric oxide (NO) produced by iNOS preferentially targets oxidative phosphorylation, and thus most ATP used for growth under nitrosative stress is derived from substrate-level phosphorylation. Zn^2+^ acquired through the ABC-type high affinity zinc transport system ZnuABC plays a critical role to the antinitrosative defenses of intracellular *Salmonella* by enabling fructose bisphosphate aldolase enzymatic activity in glycolysis. The utilization of zinc in multiple cellular processes also adds to the antinitrosative defenses of *Salmonella*.

The widespread utilization of zinc in multiple metabolic pathways may explain why Δ*znuB Salmonella* exhibits growth delays in all carbon sources tested. Our investigations have demonstrated that the ZnuABC uptake system plays a particularly salient role in the antinitrosative defenses of *Salmonella*, by perhaps promoting DksA-mediated regulation of transcription and the enzymatic activity of RecBCD DNA repair proteins [13, 40, 43]. *Salmonella* infection results in elevated levels of free zinc in macrophages, which is then readily available for the needs of the pathogen, but also undermines production of reactive oxygen and nitrogen species [44]. Despite the widespread utilization of zinc in the cell, a sizable component of the antinitrosative defenses associated with zinc uptake appears to be dependent on glycolysis. We noticed that Δ*znuB Salmonella* is particularly sensitive to the antimicrobial actions of NO when grown in glucose. The high demand for zinc in glycolysis likely reflects usage of zinc by class II fructose bisphosphate aldolase. Poor glycolytic activity might predispose Δ*znuABC* mutants to NO, an idea that is independently supported by the attenuation of Δ*pfkAB Salmonella* in mice expressing a functional iNOS hemoprotein. Glycolysis allows intracellular *Salmonella* to use the glucose available in macrophages [28], satisfying the requirements for carbon. In addition, the attenuation of Δ*pykAF* Δ*ackA* Δ*pta Salmonella* indicates that glycolysis protects against nitrosative stress by promoting ATP synthesis in substrate-level phosphorylation (**Fig 7**). Glycolysis could also boost antinitrosative defenses by balancing redox away from the electron transport chain, as has been demonstrated in *S*. *aureus* [45].

ATP generated by oxidative phosphorylation contributes very little to the antinitrosative defenses of *Salmonella* (**Fig 7**). Cytochrome *bd* and cytochrome *bo* are among the most sensitive targets of NO [46]. Nitrosylation of terminal cytochromes of the electron transport chain has a devastating effect on the ATP synthesized by oxidative phosphorylation [47]. Additionally, aconitase, pyruvate dehydrogenase, and α-ketoglutarate dehydrogenase are all sensitive to NO [10, 48], thereby not only limiting the ATP synthesized in the TCA cycle but also preventing the generation of NADH reducing power that fuels oxidative phosphorylation. Although *Salmonella* does not seem to strongly rely on oxidative phosphorylation to overcome nitrosative stress, Δ*atpB* and Δ*nuo* Δ*ndh* mutants lacking ATP synthase and NADH dehydrogenases, respectively, are highly attenuated. These phenotypes may be explained by the fact that the electron transport chain is critical for maintaining redox balance, uptaking and effluxing substrates across the cytoplasmic membrane, and driving protein folding in the cell envelope [49]. The ATP synthase working in reverse can also be a important aspect of the proton motive force [50].

As shown by others in *Borrelia burgdorfori* [12], fructose bisphosphate aldolase was found to be inactivated in *Salmonella* after NO treatment. However, fructose-bisphosphate aldolase enzymatic activity appears to be more resistant to NO toxicity than terminal cytochromes of the electron transport chain [9, 46, 51]. The high sensitivity of the electron transport chain and TCA cycle to nitrosative stress provides a reasonable explanation for the capital importance of substrate-level phosphorylation in maintaining cellular energetics of *Salmonella* undergoing nitrosative stress. The excellent recovery of *Salmonella* from nitrosative stress in the presence of both glucose and casamino acids could be partially explained by the retention of much of the ATP pool. Amino acids may also promote the recovery of *Salmonella* from NO by relieving the functional auxotrophies for branch chain amino acids, lysine and methionine that follow damage of dihydroxy-acid dehydratase and lipoamide-dependent lipoamide dehydrogenase by reactive nitrogen species [10, 11].

In summary, the ability of *Salmonella* to simultaneously exploit different carbon sources during its intracellular life-cycle [28] and diversification of metabolic pathways used to synthesize ATP may underlie the remarkable resistance of *Salmonella* to NO generated by the innate response of macrophages [7, 23]. Our investigations demonstrate that zinc allows for metabolic flexibility during nitrosative stress by allowing for proper fructose bisphosphate aldolase activity and glycolysis.

## Methods

### Ethics statement

All methods and experimental procedures were carried out in accordance to protocols approved by the University of Colorado School of Medicine (UCSOM) Institutional Biosafety Committee, authorization number 01–028. Mouse experiments were performed at Animal Care Facility of the UCSOM in accordance to the guidelines established by the UCSOM Institutional Animal Care and Use Committee (IACUC) protocol # 56413(07)1E.

### Bacterial strains

*Salmonella enterica* serovar Typhimurium strain ATCC 14028s and its mutant derivatives were used in these studies (**S7 Table**). In-frame deletion mutations were constructed using the λ Red recombinase system as originally described [52–54]. Briefly, the *kan* gene from pKD4 or pKD13, or the *cat* gene from pKD3, were PCR amplified using primers with 40–45 bases of overhang on the 5’ end that were homologous to the 40–45 bases following the ATG and the 40–45 bases preceding the stop codon of the gene of interest that was to be deleted. These linear DNA segments were gel purified, and electroporated into *Salmonella* expressing the λ Red recombinase from the plasmid pKD46 or pTP233. Transformants were selected on either LB (Luria Bertoni) kanamycin (50 μg/mL) or LB chloramphenicol (20 μg/mL) plates. Mutations were verified by PCR and were then phage (P22) transduced into 14028s wild-type *Salmonella*. Transduced colonies were selected on antibiotic plates, were confirmed by PCR, and were then cured of phage contamination. Primers used to generate and check these mutations are listed in **S7 Table**.

### Construction of the Tn-seq *Salmonella* library

Construction of the barcoded Tn-seq library in *S*. *enterica* sv Typhimurium 14028s has been described in detail elsewhere [55]. In brief, EZ-Tn5 <KAN-2> (http://www.lucigen.com) was modified to introduce an N_18_ barcode adjacent an Illumina Read 1 sequence. A library of over 230,000 different insertion mutants was constructed by mixing transposase and barcoded construct and subsequent electroporation into electrocompetent bacterial cells. The barcode associated with each unique Tn5 insertion position was determined by Illumina sequencing of PCR-amplified flanking regions, as described [55].

### Growth conditions

Strains and mutants were maintained in LB broth in the presence of 50 μg/mL kanamycin or 20 μg/mL chloramphenicol, as needed. When indicated, *Salmonella* were grown in MOPS minimal media (MOPS) [56], supplemented with either 0.4% D-glucose, 0.4% casamino acids, or 0.4% D-glucose and 0.4% casamino acids. *Salmonella* were also grown in E salts media [57] supplemented with either 0.4% D-glucose (EG), 0.1% casamino acids (ECA), or 0.4% D-glucose and 0.1% casamino acids (EGCA). When indicated, *Salmonella* were grown in N9 media (100 mM Tris-HCl, pH 7.6, 5 mM KCl, 7.5 mM (NH_4_)SO_4_, 1 mM KH_2_PO_4_, 38 mM glycerol, and 0.1% casamino acids). All cultures were grown aerobically in incubators shaking vigorously at 37°C. Chemicals were purchased from Sigma-Aldrich (St. Louis, MO) or Thermo Fisher Scientific (Hampton, NH).

### NO recovery assays

*Salmonella* grown in MOPS minimal media to an OD_600_ of 0.1–0.2 were challenged with either 750 μM spermine NONOate, 5 mM GSNO or 2.5 or 5 mM DETA NONOate (Cayman Chemical Company, Ann Arbor, MI). Growth was recorded by measuring the OD_600_ of a 200 μL sample every 30 or 60 mins in a 96-well plate. The cultures’ doubling times of the initial OD_600_ were calculated by exponential regression. Alternatively, 200 μL of overnight culture diluted 1:1000 into fresh media were seeded into honeycomb microplates and treated with either the polyamine diethylenetriamine (DETA) or DETA NONOate, and OD_600_ was recorded every 15 min for up to 40 h in a Bioscreen C plate reader (Growthcurves USA, Piscataway, NJ). Doubling times were calculated according to the equation DT = ln(2)/r, where r is the growth rate as calculated by regression analysis. The time at which the cultures reached half of their maximum growth (T = ½ OD_600_Max), a method to calculate the growth delay [10], was calculated by exponential regression.

### Screen conditions

Three different NO donors with different chemical properties were selected for our screen. Spermine NONOate spontaneously releases 2 molecules of NO per parent molecule with a half-life of 39 minutes at pH 7.4, 37°C. The NO donor DETA NONOate decays with a half-life of 20 h at pH 7.4, 37°C, producing a long and sustained flux of NO for the duration of the experiment [58]. GSNO can homolytically release NO, or heterolytically transfer an NO^+^-like species to redox active thiol groups in cysteine residues. The transposon library was challenged with these NO donors in early exponential phase in MOPS minimal media supplemented with either glucose, casamino acids, or glucose and casamino acids. The resulting populations were compared to the library grown in media alone. More specifically, the *Salmonella* Tn5 library was grown for 20 h in LB broth at 37°C with vigorous shaking. An aliquot of the culture was stocked in 10% glycerol at -80°C as the input, and another aliquot was diluted 1:100 into 10 mL of MOPS minimal media supplemented with glucose, casamino acids, or glucose and casamino acids. The cultures were grown for 2.5–4 h to an approximate OD_600_ of 0.2. The cultures were split into 2 mL aliquots and were either left untreated or were challenged with either 750 μM spermine NONOate, 5 mM GSNO, or 5 mM DETA NONOate. After 20 h of culture, the resulting populations were stocked in 10% glycerol at -80°C. The procedure was performed in biological triplicates on 3 different days. We analyzed two of those biological replicates by deep-sequencing.

Sequencing and analysis of the barcoded input and output Tn libraries was performed as described in detail in de Moraes *et al* [55]. Briefly, bacterial pellets representing approximately 5 x 10^7^ bacteria were washed three times in water followed by proteinase K digestion, enzyme inactivation, and nested PCR to amplify the N_18_ barcode region and add sample- and experiment-specific N_8_ indices. Different samples, with different indexes, were pooled and QIAquick purified (Qiagen, Hilden, Germany), followed by Illumina sequencing with standard primers. The first 18 bases in each sequencing read, which represented the unique N_18_ tag for each Tn5 mutant, were extracted, and the abundance of these 18-mers was calculated using custom perl scripts.

### Statistical analysis of the *Salmonella* Tn-seq library screen

Detailed methods of how the location and sequence of N_18_ barcode tag flanked by conserved priming sites for each Tn5 insertion mutant were identified are reported elsewhere [55]. Abundances of all mutants within each annotated feature were summed up which resulted into a data matrix where rows were annotated features and columns were samples. This data matrix was used to generate log_2_ ratios as compared to either input library or library passaged in control media (without stress inducing agents) using edgeR [59].

### Fructose bisphosphate aldolase assay

Fructose-1,6,-bisphosphate aldolase activity was assayed as previously described [12]. Briefly, wild-type and Δ*znuB Salmonella* were grown in EG minimal media to an OD_600_ of 0.4–1.0. Where indicated, the medium was supplemented with 5 μM ZnCl_2_. Some of the cultures were treated with 750 μM spermine NONOate for 5 min. Cell pellets were stored at -80°C until assayed. The pellets were resuspended in 500 μL of 100 mM Tris, pH 7.2 and the bacterial cells were lysed by sonication on ice. The specimens were centrifuged at 13,000 RPM for 15 min at 4°C. Lysates were diluted to approximately 5 mg/mL of protein as measured by A_280_ readings. Some of the specimens were treated with with 100 μM of the zinc chelator N,N,N’,N’-tetrakis(2-pyridinylmethil)-1,2-ethanediamine (TPEN) at room temperature for 30 min. Aliquots of 100 μL of lysate were added to 700 μL of 100 mM Tris, pH 7.2, 100 μL of 4 mM NADH in 100 mM Tris, pH 7.2, 100 μL of 58 mM fructose-1,6-bisphosphate trisodium salt in water, 2.5 U of type X triosephosphate isomerase from rabbit tissue and 1.5 U of type I 3-phospho-glyceraldehyde dehydrogenase, from rabbit muscle (Sigma-Aldrich, St. Louis, MO). Specimens were mixed well and four 200 μL samples were pipetted into a 96-well plate. The consumption of NADH was measured by recording the A_340_ every 20 sec for 10 min. The ΔA_340_/min was generated by linear regression and technical replicates were averaged and normalized to the protein in the sample. All activities were normalized to untreated samples gathered from wild-type *Salmonella*.

### Intracellular replication

J774A1 murine macrophage-like cells (ATCC TIB67) were maintained at 37°C, 5% CO_2_ in RPMI^+^ media (RPMI media supplemented with 2 mM L-glutamate, 1 mM sodium pyruvate, 15 mM HEPES buffer, 10% fetal bovine serum, and 100 U/mL Penicillin-Streptomycin). Twenty hours prior to infection, J774 cells were plated at 10^5^ cells per well in a 96-well plate in 100 μL of media. *Salmonella* cultures were grown for 20 h at 37°C with vigorous shaking. Overnight bacterial cultures were diluted to approximately 2x10^6^ CFU/mL in macrophage media without Pen/Strep. J774 cells were washed with prewarmed RPMI^+^ media, and were challenged with *Salmonella* at an MOI of 2. The plates were centrifuged at 4000 RPM for 1 min at room temperature, and then incubated at 37°C, 5% CO_2_. After 25 min, the culture media was removed and fresh RPMI^+^ media containing 50 μg/mL of gentamicin was added to the macrophages at 37°C and, after 1h, the media was replaced with media containing 10 μg/mL gentamicin. One and seventeen h later (T = 2 and 18 h of infection), the media was removed and the macrophages were lysed with 0.25% (w/v) deoxycholic acid prepared in PBS. The bacterial burdens were quantified after 10-fold serial dilutions on LB agar plates. Fold-replication was calculated by normalizing the CFU/mL at T = 18 h to the CFU/mL at T = 2 h. When applicable, nitrite in the culture supernatants was assayed with the Griess reaction as previously described [60].

### Survival in primary macrophages

The intracellular survival of wild-type and mutant *Salmonella* was tested in periodate-elicited macrophages as described [61]. Macrophages from C57BL/6 and iNOS^-/-^ [62] mice were infected at MOI of 2, and survival was determined after 16–20 h of challenge.

### Murine infections

These following studies were approved by the Institutional Animal Care and Use Committee at the University of Colorado—Denver Anschutz Medical Campus. Eight to ten week-old C57BL/6 and congenic *iNOS*^*-/-*^ mice were infected *i*.*p*. with 100–200 CFU of the indicated *Salmonella* strains. Mouse survival was monitored over time.

### Thin layer chromatography estimations of ATP pools

Wild-type *Salmonella* were grown for 20 h in MOPS supplemented with 2 mM HK_2_PO_4_ and either glucose, casamino acids, or glucose and casamino acids. The overnight cultures were diluted 1:100 into MOPS supplemented with the same carbon source and 0.4 mM HK_2_PO_4_. The bacteria were grown to an OD_600_ of 0.2 and then labeled with 10 μCi of ^32^P orthophosphate in 1 mL aliquots. After approximately 2.5 generations, at an OD_600_ of 0.5, cultures were either left untreated or were challenged for 5 min with 750 μM spermine NONOate. One mL cultures were mixed with 0.4 mL of ice-cold 50% formic acid and samples were put on ice for at least 15 min prior to centrifugation for 5 min at 13,000 RPM. 10 μL of lysates were spotted along the bottom of polyethyleneimine-cellulose TLC plates (EMD Millipore, Darmstadt, Germany). The TLC plates were air-dried and then placed into a chromatography chamber containing either 1.25 or 0.9 M KH_2_PO_4_, pH 3.4. The solvent system was allowed to migrate 15–19 cm up the 20 cm TLC plate. The plates were air-dried, placed inside plastic wrap, and placed on a phosphorscreen overnight. The following day the screens were scanned with a phosphorimager. Images were cropped and brightness and contrast were adjusted in Photoshop 11.0.

### Firefly luciferase estimations of ATP pools

Intracellular pools of ATP were calculated with the luciferase-based ATP Determination Kit (Molecular Probes, Eugene, OR) as instructed by the manufacturer with a few minor adjustments. Briefly, wild-type *Salmonella* grown to an OD_600_ of 0.2–0.5 were either left untreated or challenged with 750 μM spermine NONOate for 5 min. Samples (0.5 mL culture) were thoroughly mixed with 0.6 mL freshly prepared, ice-cold 380 mM formic acid and 17 mM EDTA, and small samples were taken to quantify bacterial density. Formic acid-EDTA samples were saved at -80°C until assayed. Specimens were centrifuged for 1 min at 13,000 RPM, and the supernatants were diluted 25-fold into 100 mM TES buffer, pH 7.4 to neutralize the formic acid. Ten μL of samples or solutions with known concentrations of ATP were mixed with 90 μL of reaction master mix (10 mL; 8.9 mL water, 500 μL of 20X buffer, 500 μL of 10 mM D-luciferin, 100 μL of 100 mM DTT, and 2.5 μL of 5 mg/mL firefly luciferase) in white 96-well plates, and luminescence was recorded in a Lmax 1.1L machine (Molecular Devices, San Jose, CA), and the data analyzed using the SOFTmax Pro software. Concentrations of ATP in the culture were generated using linear regression of ATP standards. Intracellular ATP concentrations of the original samples were calculated using the CFU/mL counts assuming a cell volume of 1 fL.

### Statistical analysis

Statistical analyses were performed using GraphPad Prism 5.0b Software. One-way and two-way ANOVA, t-tests and logrank tests were used. Results were determined to be significant when *p* < 0.05.

## Supporting information

S1 Table All genes. (XLSX)

S2 Table Genes for Venn diagram. (XLSX)

S3 Table Shared genes. (XLSX)

S4 Table NO-related genes. (XLSX)

S5 Table Zinc-related genes. (XLSX)

S6 Table Bacterial strains. (DOCX)

S7 Table Primers. (DOCX)

S1 Fig Effect of carbon source on the recovery of *Salmonella* from S-nitrosylglutathione (GSNO) and diethlyenetriamine NONOate (dNO). (A) The time (min) required for *Salmonella* to double the initial culture density was calculated for bacteria growing in MOPS minimal media supplemented with either glucose (GLC), casamino acids (CAA), or glucose and casamino acids (GLC + CAA) with or without 750 μM spermine NONOate challenge (N = 4, mean ± S.E.M.). *, **, ***, *p* < 0.05, 0.01, 0.001, respectively, as determined by one-way ANOVA. *Salmonella* grown in MOPS minimal media supplemented with either GLC, CAA, or GLU + CAA were either untreated (ctrl) or challenged with 5 mM of either GSNO (B) or dNO (C). Bacterial growth was estimated by following OD_600_ measurements every hour. (D) Growth of wild-type (WT) and Δ*pfkAB Salmonella* in EG minimal media or MOPS minimal media supplemented with glycerol (N = 12, mean).
(DOCX)

S2 Fig Recovery of *Salmonella* deficient in Zn^2+^ metabolism from nitrosative stress. Delay in growth (A) and rate of growth (B) of wild-type (WT) and Δ*znuB Salmonella* in EG, ECA, and EGCA minimal media. Select samples were supplemented with 5 μM ZnCl_2_ or challenged with 1 mM diethylenetriamine (DETA) or DETA NONOate (dNO) (N = 5 or 10, mean ± S.E.M.) (C) Growth of Δ*znuB Salmonella* complemented with a wild-type *znuB* gene in EG media. Selected samples were treated with 1 mM dNO. (D) Growth of WT and Δ*zur*, Δ*znuA*, Δ*znuB*, and Δ*znuC Salmonella* in EGCA minimal media challenged with either 5 mM DETA or 5 mM dNO (N = 10, mean). Growth rates (E) and delays (F) were calculated by exponential regression (N = 10, mean ± S.E.M.). (G, H) Replication of *Salmonella* after 16–20 h of culture in J774 cells. Selected samples were treated with 500 μM L-NIL. (I, K) The concentration of nitrite in the supernatants of *Salmonella*-infected macrophages was estimated by the Griess reaction (N = 16, mean ± S.E.M.). *, **, *** *p* < 0.05, 0.01, 0.001, respectively, as determined by two-way ANOVA or *t*-test. (J) Effect of AG or L-NIL on the growth of *Salmonella* in EG media.
(DOCX)

S3 Fig Nitrotyrosine formation in NO-treated *Salmonella*. *Salmonella* were grown in EG minimal media to OD_600_ of 0.4 at 37°C with shaking. The bacteria were lysed by sonication and the specimens were tested for the presence of nitrotyrosine residues by Western blotting as described [1]. Where indicated (+), the bacteria were treated with 500 μM spermine NONOate for 30 min prior to sonication. The blot is representative of 2 independent samples. *, proteins nonspecific labeled by the anti-nitrotyrosine antibodies. Arrows indicate proteins bearing nitrotyrosine residues.
(DOCX)

S4 Fig Growth and recovery of glycolysis and ATP synthesizing mutants. Growth delay (A) and rate (B) of wild-type (WT) and Δ*pfkAB Salmonella* in LB broth challenged with 5 mM DETA NONOate were calculated by exponential regression (N = 5, mean ± S.E.M.). (C) NO production from J774 cells infected with *Salmonella* was estimated by the Griess reaction (N = 4 or 8, mean ± S.E.M.). Replication of Δ*pfkAB Salmonella* complemented with *pfkA* or *pfkB* genes in J774 cells (D) or EG media +/- 1 mM dNO (E). (F) NO production from J774 cells infected with WT, Δ*atpB*, and Δ*ackA* Δ*pta Salmonella* was estimated by the Griess reaction (N = 4 or 8, mean ± S.E.M.). *, **, ***; *p* < 0.05, 0.01, 0.001, respectively, as determined by two-way ANOVA.
(DOCX)

S5 Fig ATP pools in Δ*atpB Salmonella*. (A) ATP pools in WT, Δ*atpB*, and Δ*ackA* Δ*pta Salmonella* was estimated with firefly luciferase and normalized to culture density (N = 6, mean ± S.E.M.). Selected cultures were treated with 750 μM spermine NONOate (sNO). (B) Effect of 750 μM spermine NONOate (sNO) on the growth of *Salmonella*. The Δ*pykAF* Δ*ackA* Δ*pta* mutant was complemented with the low copy number plasmid pWSK29 harboring the *ackA pta* operon (pACKPTA). (C) Intracellular growth of the indicated *Salmonella* strains after 16h of culture in J774 cells. (D) Production of nitrite by *Salmonella*-infected, periodate-elicited macrophages was estimated spectrophotometrically by the Griess reaction.
(DOCX)
