# Supplementary material for: Zinc-dependent substrate-level phosphorylation powers Salmonella growth under nitrosative stress of the innate host response
Source: PLoS Pathog. 2018 Oct 26;14(10):e1007388. doi: 10.1371/journal.ppat.1007388 (PMC6221366; doi:10.1371/journal.ppat.1007388)
Supplement: S6 Table — (DOCX) [file ppat.1007388.s006.docx]

**S6 Table: Bacterial strains**

| Strains | Genotype | Source |
| --- | --- | --- |
| *Salmonella enterica* serovar Typhimurium, 14028s | Wild-type | ATCC |
| AV14170 | Δ*znuA*::*kan* | This study |
| AV14171 | Δ*znuB*::*kan* | This study |
| AV14172 | Δ*znuC*::*kan* | This study |
| AV14174 | Δ*zur*::*kan* | This study |
| AV17122 | Δ*atpB*::cat | This study |
| AV09580 | Δ*pfkA*::FRT Δ*pfkB*::*kan* | This study |
| AV09461 | Δ*gltA*::*kan* | This study |
| AV10193 | Δ*acnA*::FRT Δ*acnB*::*kan* | This study |
| AV08257 | Δ*icdA*::*FRT* | This study |
| AV08241 | Δ*sucAB*::*FRT* | This study |
| AV09459 | Δ*sucCD*::kan | This study |
| AV09458 | Δ*sdhAB*::kan | This study |
| AV09573 | Δ*fumAC*::*FRT* | This study |
| AV08212 | Δ*mdh*::*FRT* | This study |
| AV09460 | Δ*glk*::*kan* | This study |
| AV08218 | Δ*pgi*::*FRT* | This study |
| AV09578 | Δ*pykA*::FRT Δ*pykF*::*kan* | This study |
| AV10379 | Δ*ackA*::FRT Δ*pta*::*kan* | This study |
| AV18115 | Δ*ackA* *pta*::*cm* | This study |
| AV18116 | Δ*pykA* Δ*pykF* Δ*ackA* *pta*::*cm* | This study |
| AV18226 | pWSK29::*znuB* | This study |
| AV18227 | pWSK29::*pykA* | This study |
| AV18228 | pWSK29::*pykF* | This study |
| AV18229 | Δ*znuB::Km* pWSK29::*znuB* | This study |
| AV18232 | pBAD18::*fbaA*::*Pn* | This study |
| AV18233 | pBAD18::*fbaB*::*Pn* | This study |
| AV18234 | Δ*znuB*::*Km* pBAD18::*fbaA*::*Pn* | This study |
| AV18235 | Δ*znuB*::*Km* pBAD18::*fbaB*::*Pn* | This study |
| AV18236 | pWSK29::*ackA pta*::*Pn* | This study |
| AV18237 | pWSK29::*pfkA*::Pn | This study |
| AV18238 | pWSK29::*pfkB*::Pn | This study |
| AV18239 | Δ*pfkA* Δ*pfkB* pWSK29::*pfkA*::*Pn* | This study |
| AV18240 | Δ*pfkA* Δ*pfkB* pWSK29::*pfkB*::*Pn* | This study |
| AV18241 | Δ*ackA* *pta*::*Cm* Δ*pykA* Δ*pykF*::*Km* pWSK29::*ackA* *pta*::*pn* | This study |
