# Supplementary material for: Zinc-dependent substrate-level phosphorylation powers Salmonella growth under nitrosative stress of the innate host response
Source: PLoS Pathog. 2018 Oct 26;14(10):e1007388. doi: 10.1371/journal.ppat.1007388 (PMC6221366; doi:10.1371/journal.ppat.1007388)
Supplement: S7 Table — (DOCX) [file ppat.1007388.s007.docx]

**S7 Table: Primers**

| **Gene** |  | **Primer Sequence (5' to 3')** |
| --- | --- | --- |
| *znuA* | F | TTTAGGCTTGCAGAAGTGATAGAATGTTATAATATCACATTTCACGTGCAGGCTGGAGCTGCTTC |
|  | R | GTATTCACTTCCTCGTTAATCTCCTTTCAGGCAGCTCGCATACTGCATATGAATATCCTCCTTAG |
| *znuB* | F | GGGAAATGGTCACTCATGATTGAATTATTACTGCCCGGCTGGCTGGTGCAGGCTGGAGCTGCTTC |
|  | R | CCGGGCAACACACGCTTAGCTTGCCTGCTTTTTCATCATACTGAACATATGAATATCCTCCTTAG |
| *znuC* | F | CATCGTAATGAATGTGTGAAATGTGATATTATAACATTCTATCACGTGCAGGCTGGAGCTGCTTC |
|  | R | GCAGTAATAATTCAATCATGAGTGACCATTTCCCCGGCGCAGTACCATATGAATATCCTCCTTAG |
| *zur* | F | CATTCGAGGTGCTACATGGAAAAGACCACAACGCAAGAGTTACTGGTGCAGGCTGGAGCTGCTTC |
|  | R | CGTTTTCCGTTCAGGCTAACGTGGTTTTTTCTTCACCAGCACCGACATATGAATATCCTCCTTAG |
| *atpB* | F | CCAGTTTGTTTCAGTTAAAACGTAGTAGTGTTGGTAAATATGAATATCCTCCTTAGTT |
|  | R | GTGGTTCAGATACTGGCGCCGGCTGTAATTAACAACAGTGTAGGCTGGAGCTGCTTC |
| *pfkA* | *F* | CAATAGATTTCATTTTGCATTCCAAAGTTCAGAGGTAGTCGTGTAGGCTGGAGCTGCTTC |
|  | *R* | AGGCCTGATAAGCGTAGCGCCATCAGGCGCGCAAAAACAAATTCCGGGGATCCGTCGACC |
| *pfkB* | *F* | ATTAAGTGCCAGACTGAAATCAGCCTAACAGGAGGTAACGGTGTAGGCTGGAGCTGCTTC |
|  | *R* | AACCGATTTTCCGTTATCCCCCTCGGCGAGGGGGAAACGAATTCCGGGGATCCGTCGACC |
| *gltA* | F | TCCGGCAGTCTTAAGCAATAAGGCGCTAAGGAGACCGTAAGTGTAGGCTGGAGCTGCTTC |
|  | R | GTACCGGATGGCGAGGGTTGCGCATCCGGTGTCAAATTCATTCCGGGGATCCGTCGACC |
| *acnA* | F | agaatcagggtaacgcaaccctgtcatttaaggaggagctgtgtaggctggagctgcttc |
|  | R | gcgcgggcctgcctgtttacggcaggcccgataaacggctattccggggatccgtcgacc |
| *acnB* | F | aagcgccgcattatgacaatgagagcgaggagatatcgtcgtgtaggctggagctgcttc |
|  | R | atgaataaaaagggggcaattagcccccttaaaatgtaaaattccggggatccgtcgacc |
| *icdA* | F | ggtaagaagatcaccctgcaaaacggcaaactcaacgttcgtgtaggctggagctgcttc |
|  | R | ctcactacatttcagcagcttagcgccttccatcaggcgtattccggggatccgtcgacc |
| *sucAB* | F | acctctctggttcgaatcagagctggatagaacagctctagtgtaggctggagctgcttc |
|  | R | accctaccgccggagaggcggaggccgggcgacctgcataattccggggatccgtcgacc |
| *sucCD* | *F* | GGTCTAAAGATAACGATTACCTGAAGGATGGACAGAACACGTGTAGGCTGGAGCTGCTTC |
|  | *R* | GAAAACGGACATTTATCTGTTCCCGCAGGAACAGCGAGTTATTCCGGGGATCCGTCGACC |
| *sdhAB* | *F* | TTTACGTCATCTATGGATTTGTTGTGGTGTGGGGTGTGTAGTGTAGGCTGGAGCTGCTT |
|  | *R* | ATAAGACTGTACGTCGCCATCCGGCAACCACTACAACTACATTCCGGGGATCCGTCGACC |
| *fumAC* | F | GCCCAGAGAATAACCATACCGAGCGGTAAGTGAGAGCACAGTGTAGGCTGGAGCTGCTTC |
|  | R | TGCGACTGATTATTCCGCGACTGCACCTGTATGTGGCGGAATTCCGGGGATCCGTCGACC |
| *mdh* | F | Ggtatcggtcaggcgctggcattacttttaaaaaaccaacgtgtaggctggagctgcttc |
|  | R | Aatatcttttttcagcgtatccagcatagcgtccagcgaaattccggggatccgtcgacc |
| *glk* | F | TGACAAAGACTTATTTTGACTTTAGCGGAGCAGTAGAAGAGTGTAGGCTGGAGCTGCTTC |
|  | R | CTTTTGTAGGCCGGATAAGGCGTTTATGCCACCATCTGGAATTCCGGGGATCCGTCGACC |
| *pgi* | F | Ccaacgcagacttctgcctggcaggcgctccagaaacactgtgtaggctggagctgcttc |
|  | R | Tttataacggttaatcagaccgttagtagagctatcatggattccggggatccgtcgacc |
| *pykA* | F | Ttcacgcaacaccaagttgttttagtcaacggagtattacgtgtaggctggagctgcttc |
|  | R | Aaccctgtactacctgatgaacaggcgtgggggagttttaattccggggatccgtcgacc |
| *pykF* | F | Agatttccatatcctcctcaacttaaagactaagactgtcgtgtaggctggagctgcttc |
|  | R | Aagggcgcttttttaaacaaattaattcacacaacaattaattccggggatccgtcgacc |
| *ackA pta* | F  R | tttagccacgtatcataaataggtacttccatggtgtaggctggagctgcttc  ggcgttcacgccgccatccggcattagcttttactgttacatatgaatatcctcctt |
| pWSK29::znuB | F  R | \| Ccgctcgagtgctctgcttaacaggcctcagttg \| \| --- \| \| cgcgaattcTTAGCTTGCCTGCTTTTTCATCATACTGAAG \| |
| pBAD18::*fbaA* | F  R | \| CCGGAgcTCTGATAGCGAAGCGTTTTCCATCGCTG \| \| --- \| \| TGCTCTAGATTACAGAACGTCAATCGCGTTCAGTTC \| |
| pBAD18::*fbaB* | F  R | \| CCGGAgcTCaaccactccacgaacatcatgaacgac \| \| --- \| \| TGCTCTAGATCAGGCGATGGTGACTTTGCTATCC \| |
| pWSK29::*ackApta* | F  R | \| Ccgctcgagaggtatcctgtgtcggcctgtc \| \| --- \| \| cgcgaattcTTACTGCTGCTGCTGAGAAGCCTGGATCGCCGTC \| |
| pWSK29::*pfkA* | F  R | \| Ccgctcgagtacagcgttttccgggttcagatg \| \| --- \| \| actGAGCTCTCAGTACAGTTTTTTCGCGCACTCCAT \| |
| pWSK29::*pfkB* | F  R | \| Ccgctcgagaaaaaagagaccacgacggtcc \| \| --- \| \| cgcgaattcTTATTGCGCGGAAAGATAGGCGTA \| |
