# Supplementary figures and images for: Zinc-dependent substrate-level phosphorylation powers Salmonella growth under nitrosative stress of the innate host response
Source: PLoS Pathog. 2018 Oct 26;14(10):e1007388. doi: 10.1371/journal.ppat.1007388 (PMC6221366; doi:10.1371/journal.ppat.1007388)

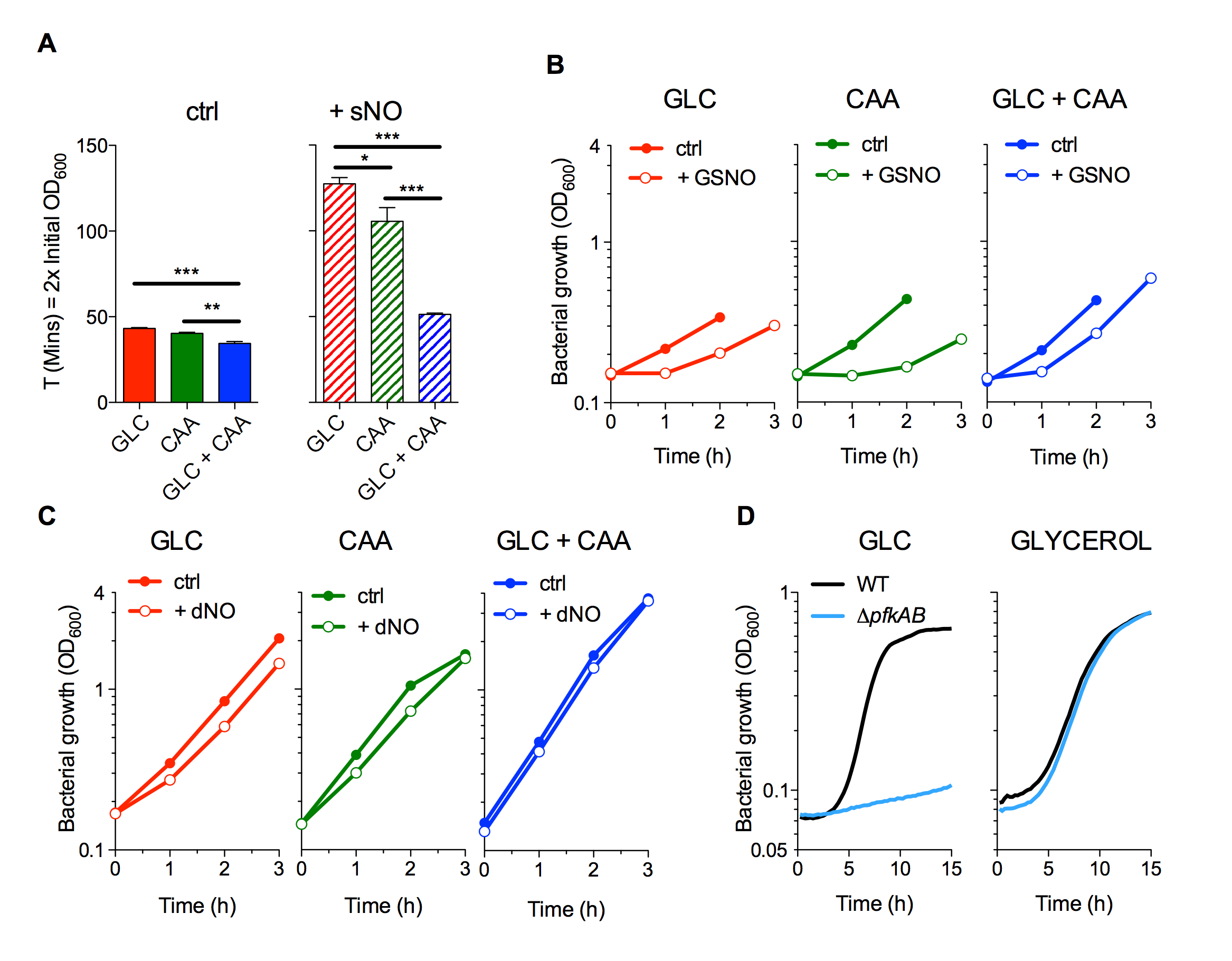

Supplement: S1 Fig — (A) The time (min) required for Salmonella to double the initial culture density was calculated for bacteria growing in MOPS minimal media supplemented with either glucose (GLC), casamino acids (CAA), or glucose and casamino acids (GLC + CAA) with or without 750 μM spermine NONOate challenge (N = 4, mean ± S.E.M.). *, **, ***, p < 0.05, 0.01, 0.001, respectively, as determined by one-way ANOVA. Salmonella grown in MOPS minimal media supplemented with either GLC, CAA, or GLU + CAA were either untreated (ctrl) or challenged with 5 mM of either GSNO (B) or dNO (C). Bacterial growth was estimated by following OD600 measurements every hour. (D) Growth of wild-type (WT) and ΔpfkAB Salmonella in EG minimal media or MOPS minimal media supplemented with glycerol (N = 12, mean). (DOCX) [file ppat.1007388.s008.docx]

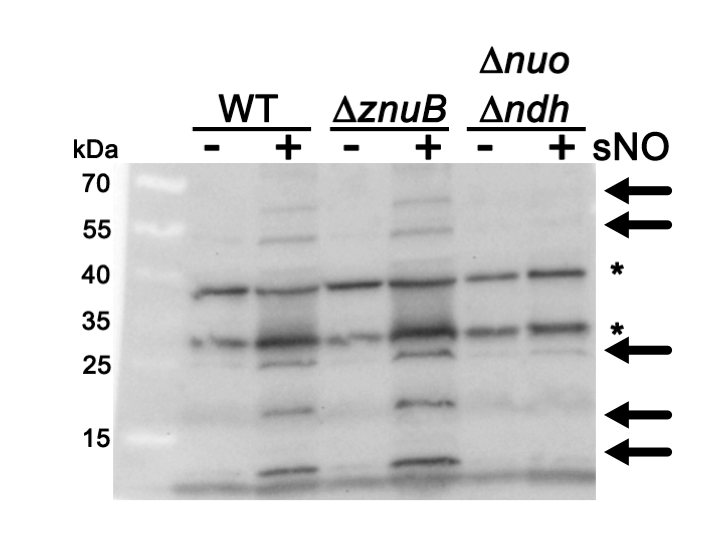

Supplement: S3 Fig — Salmonella were grown in EG minimal media to OD600 of 0.4 at 37°C with shaking. The bacteria were lysed by sonication and the specimens were tested for the presence of nitrotyrosine residues by Western blotting as described [1]. Where indicated (+), the bacteria were treated with 500 μM spermine NONOate for 30 min prior to sonication. The blot is representative of 2 independent samples. *, proteins nonspecific labeled by the anti-nitrotyrosine antibodies. Arrows indicate proteins bearing nitrotyrosine residues. (DOCX) [file ppat.1007388.s010.docx]

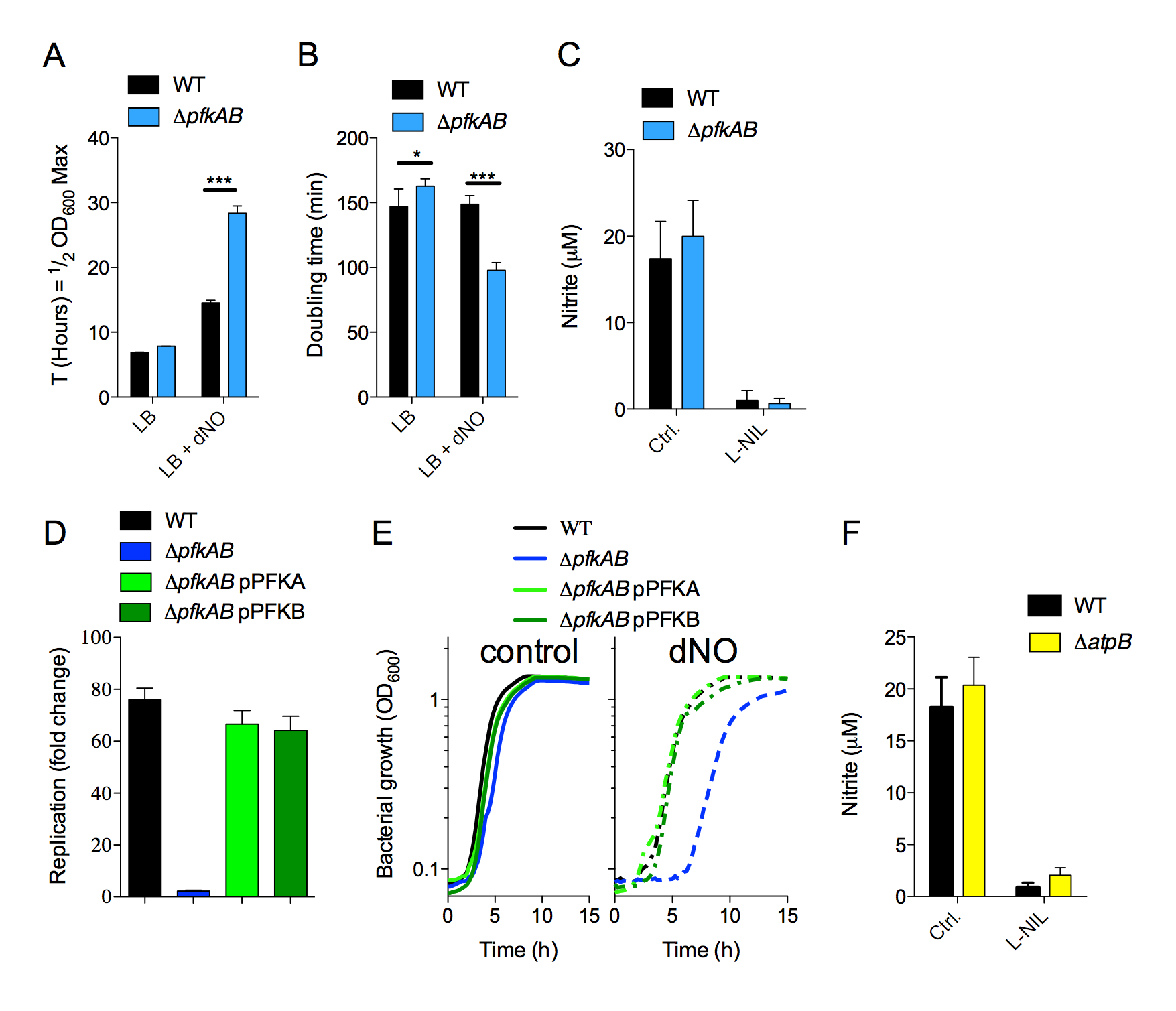

Supplement: S4 Fig — Growth delay (A) and rate (B) of wild-type (WT) and ΔpfkAB Salmonella in LB broth challenged with 5 mM DETA NONOate were calculated by exponential regression (N = 5, mean ± S.E.M.). (C) NO production from J774 cells infected with Salmonella was estimated by the Griess reaction (N = 4 or 8, mean ± S.E.M.). Replication of ΔpfkAB Salmonella complemented with pfkA or pfkB genes in J774 cells (D) or EG media +/- 1 mM dNO (E). (F) NO production from J774 cells infected with WT, ΔatpB, and ΔackA Δpta Salmonella was estimated by the Griess reaction (N = 4 or 8, mean ± S.E.M.). *, **, ***; p < 0.05, 0.01, 0.001, respectively, as determined by two-way ANOVA. (DOCX) [file ppat.1007388.s011.docx]

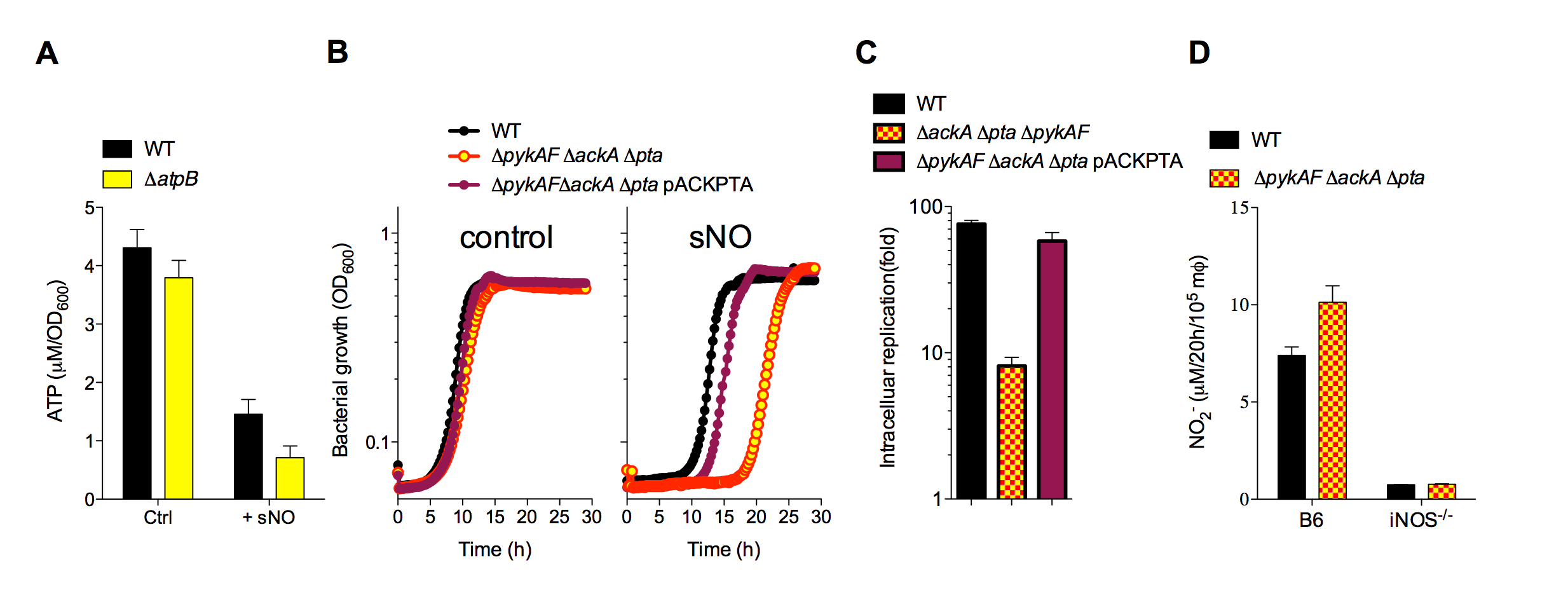

Supplement: S5 Fig — (A) ATP pools in WT, ΔatpB, and ΔackA Δpta Salmonella was estimated with firefly luciferase and normalized to culture density (N = 6, mean ± S.E.M.). Selected cultures were treated with 750 μM spermine NONOate (sNO). (B) Effect of 750 μM spermine NONOate (sNO) on the growth of Salmonella. The ΔpykAF ΔackA Δpta mutant was complemented with the low copy number plasmid pWSK29 harboring the ackA pta operon (pACKPTA). (C) Intracellular growth of the indicated Salmonella strains after 16h of culture in J774 cells. (D) Production of nitrite by Salmonella-infected, periodate-elicited macrophages was estimated spectrophotometrically by the Griess reaction. (DOCX) [file ppat.1007388.s012.docx]
