# Supplementary material for: Zinc-dependent substrate-level phosphorylation powers Salmonella growth under nitrosative stress of the innate host response
Source: PLoS Pathog. 2018 Oct 26;14(10):e1007388. doi: 10.1371/journal.ppat.1007388 (PMC6221366; doi:10.1371/journal.ppat.1007388)
Supplement: Supplementary file 9 [file ppat.1007388.s009.docx]

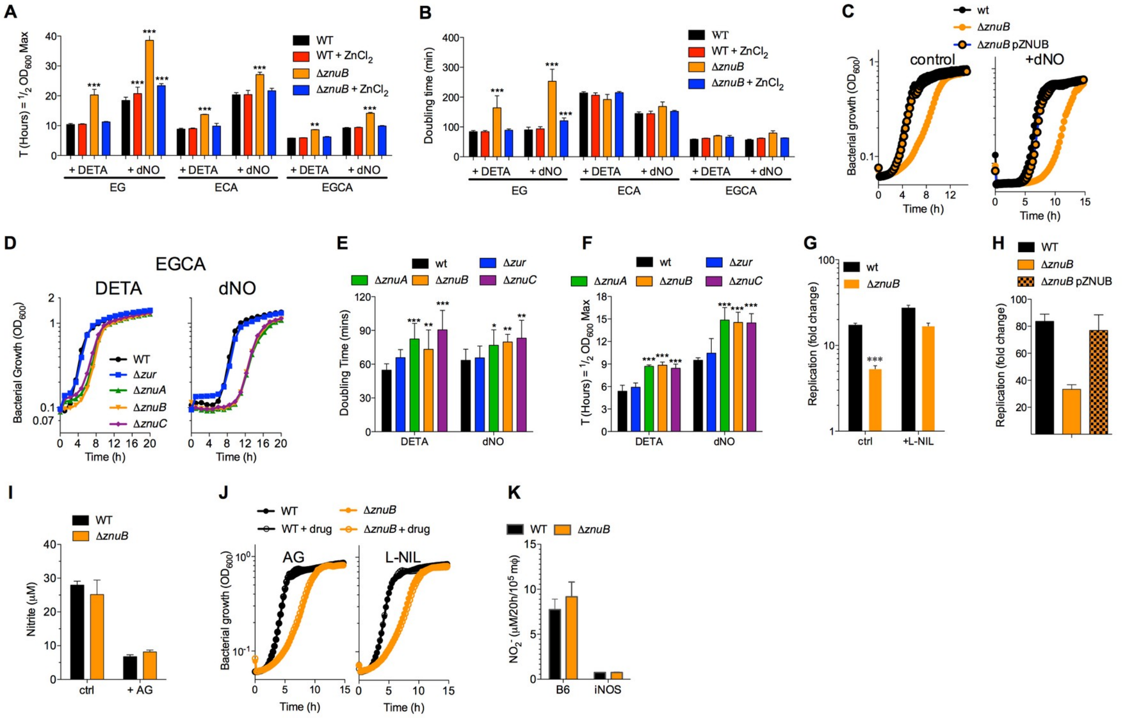

A
T (Hours) = 1/2 OD600 Max
WT
WT + ZnCl2
ΔznuB
ΔznuB + ZnCl2
+ DETA
+ dNO
EG
ECA
EGCA
B
Doubling time (min)
WT
WT + ZnCl2
ΔznuB
ΔznuB + ZnCl2
+ DETA
+ dNO
EG
ECA
EGCA
C
wt
ΔznuB
ΔznuB pZNUB
control
+dNO
Bacterial growth (OD600)
Time (h)
D
EGCA
DETA
dNO
Bacterial Growth (OD600)
WT
Δzur
ΔznuA
ΔznuB
ΔznuC
Time (h)
E
wt
Δzur
ΔznuA
ΔznuB
ΔznuC
Doubling Time (mins)
DETA
dNO
F
wt
Δzur
ΔznuA
ΔznuB
ΔznuC
T (Hours) = 1/2 OD600 Max
DETA
dNO
G
wt
ΔznuB
Replication (fold change)
ctrl
+L-NIL
H
WT
ΔznuB
ΔznuB pZNUB
Replication (fold change)
I
WT
ΔznuB
Nitrite (μM)
ctrl
+ AG
J
WT
ΔznuB
WT + drug
ΔznuB + drug
AG
L-NIL
Bacterial growth (OD600)
Time (h)
K
WT
ΔznuB
NO2- (μM20h/10^5 mφ)
B6
iNOS
